# Efficacy against pneumococcal carriage and the immunogenicity of reduced-dose (0 + 1 and 1 + 1) PCV10 and PCV13 schedules in Ho Chi Minh City, Viet Nam: a parallel, single-blind, randomised controlled trial

**DOI:** 10.1016/S1473-3099(23)00061-0

**Published:** 2023-08

**Authors:** Beth Temple, Hau Phuc Tran, Vo Thi Trang Dai, Heidi Smith-Vaughan, The VPT-II Collaborator Group, Anne Balloch, Jemima Beissbarth, Kathryn Bright, Rachel Ann Higgins, Jason Hinds, Pham Thi Hoan, Monica Larissa Nation, Cattram Duong Nguyen, Belinda Daniela Ortika, Thanh V Phan, Tran Linh Phuong, Leena Spry, Ho Nguyen Loc Thuy, Nguyen Trong Toan, Doan Y Uyen, Le Thi Tuong Vy, Paul Vincent Licciardi, Catherine Satzke, Thuong Vu Nguyen, Kim Mulholland

**Affiliations:** a Global and Tropical Health Division, Menzies School of Health Research, Charles Darwin University, Darwin, NT, Australia; b Child Health Division, Menzies School of Health Research, Charles Darwin University, Darwin, NT, Australia; c Infection and Immunity, Murdoch Children's Research Institute, Melbourne, VIC, Australia; d Department of Infectious Disease Epidemiology, London School of Hygiene & Tropical Medicine, London, UK; e Department of Disease Control and Prevention, Pasteur Institute of Ho Chi Minh City, Ho Chi Minh City, Viet Nam; f Department of Microbiology and Immunology, Pasteur Institute of Ho Chi Minh City, Ho Chi Minh City, Viet Nam; g Department of Paediatrics, University of Melbourne, Melbourne, VIC, Australia; h Department of Microbiology and Immunology, University of Melbourne at the Peter Doherty Institute for Infection and Immunity, Melbourne, VIC, Australia

## Abstract

**Background:**

Interest in reduced-dose pneumococcal conjugate vaccine (PCV) schedules is growing, but data on their ability to provide direct and indirect protection are scarce. We evaluated 1 + 1 (at 2 months and 12 months) and 0 + 1 (at 12 months) schedules of PCV10 or PCV13 in a predominately unvaccinated population.

**Methods:**

In this parallel, single-blind, randomised controlled trial, healthy infants aged 2 months were recruited from birth records in three districts in Ho Chi Minh City, Vietnam, and assigned (4:4:4:4:9) to one of five groups: PCV10 at 12 months of age (0 + 1 PCV10), PCV13 at 12 months of age (0 + 1 PCV13), PCV10 at 2 months and 12 months of age (1 + 1 PCV10), PCV13 at 2 months and 12 months of age (1 + 1 PCV13), and unvaccinated control. Outcome assessors were masked to group allocation, and the infants' caregivers and those administering vaccines were not. Nasopharyngeal swabs collected at 6 months, 12 months, 18 months, and 24 months were analysed for pneumococcal carriage. Blood samples collected from a subset of participants (200 per group) at various timepoints were analysed by ELISA and opsonophagocytic assay. The primary outcome was the efficacy of each schedule against vaccine-type carriage at 24 months, analysed by intention to treat for all those with a nasopharyngeal swab available. This trial is registered at ClinicalTrials.gov, NCT03098628.

**Findings:**

2501 infants were enrolled between March 8, 2017, and July 24, 2018 and randomly assigned to study groups (400 to 0 + 1 PCV10, 400 to 0 + 1 PCV13, 402 to 1 + 1 PCV10, 401 to 1 + 1 PCV13, and 898 to control). Analysis of the primary endpoint included 341 participants for 0 + 1 PCV10, 356 0 + 1 PCV13, 358 1 + 1 PCV10, 350 1 + 1 PCV13, and 758 control. At 24 months, a 1 + 1 PCV10 schedule reduced PCV10-type carriage by 58% (95% CI 25 to 77), a 1 + 1 PCV13 schedule reduced PCV13-type carriage by 65% (42 to 79), a 0 + 1 PCV10 schedule reduced PCV10-type carriage by 53% (17 to 73), and a 0 + 1 PCV13 schedule non-significantly reduced PCV13-type carriage by 25% (–7 to 48) compared with the unvaccinated control group. Reactogenicity and serious adverse events were similar across groups.

**Interpretation:**

A 1 + 1 PCV schedule greatly reduces vaccine-type carriage and is likely to generate substantial herd protection and provide some degree of individual protection during the first year of life. Such a schedule is suitable for mature PCV programmes or for introduction in conjunction with a comprehensive catch-up campaign, and potentially could be most effective given as a mixed regimen (PCV10 then PCV13). A 0 + 1 PCV schedule has some effect on carriage along with a reasonable immune response and could be considered for use in humanitarian crises or remote settings.

**Funding:**

Bill & Melinda Gates Foundation.

**Translation:**

For the Vietnamese translation of the abstract see Supplementary Materials section.

## Introduction

Infant vaccination with ten-valent pneumococcal conjugate vaccine (PCV10) or 13-valent pneumococcal conjugate vaccine (PCV13) is a proven strategy to protect both vaccinees (through direct protection) and the broader population (through indirect or herd protection) against disease caused by vaccine-type *Streptococcus pneumoniae*. Herd protection arises through reduced nasopharyngeal carriage of vaccine-type pneumococci among vaccinees, and consequently reducing pneumococcal transmission to unvaccinated individuals.^1^ Vaccine-type disease incidence substantially declines following PCV introduction and the importance of herd protection over individual protection therefore increases.^2^ WHO currently recommends PCV administration in a 3 + 0 (three-dose primary series with no booster) or a 2 + 1 (two-dose primary series with booster) schedule.^3^ However, schedules with fewer doses are likely to be effective for countries with mature PCV programmes and high vaccine coverage. Administering a single dose of PCV in the second year of life (0 + 1 schedule) might be sufficient to maintain herd protection,^4^ as young children are key transmitters of pneumococci.^5^ Adding an infant dose (1 + 1 schedule) would provide additional, direct protection during the first year of life and enhance the response to the booster dose. In 2020, the UK became the first country to switch to a 1 + 1 schedule (with doses at 12 weeks and 12 months of age). This decision was based on favourable post-booster immunogenicity data from a trial^6^ comparing 1 + 1 and 2 + 1 PCV13 schedules. Post-booster responses with a 1 + 1 schedule (with doses either at 6 weeks and 9 months or at 14 weeks and 9 months) of PCV10 or PCV13 have also been shown to be non-inferior to those with a 2 + 1 schedule in South Africa.^7^ Neither of these trials included carriage outcomes. Given the role of carriage in mediating herd protection, such information is of paramount importance, yet there is a paucity of data available. A previous trial in Ho Chi Minh City, Viet Nam, showed that a 1 + 1 PCV10 schedule at 2 months and 6 months reduced vaccine-type carriage and was immunogenic.^8^, ^9^ Vaccine-type carriage also reduced following a 0 + 1 PCV10 schedule in Kenyan children aged between 1 and 4 years,^10^ and following a 0 + 1 PCV10 schedule at 18-months in the earlier Ho Chi Minh City trial.^11^ Here, we report the efficacy against carriage and the immunogenicity of 1 + 1 and 0 + 1 schedules of PCV10 or PCV13 in Ho Chi Minh City, where PCV is not part of the national routine immunisation programme.

**Research in context**

**Evidence before this study**

Pneumococcal conjugate vaccines (PCVs) have been available since 2000, but an estimated 60% of the world's children are unvaccinated. Vaccination strategies providing greater accessibility and affordability are urgently needed. Reduced-dose schedules offer one such strategy. A single dose at or after 12 months of age (0 + 1 schedule) might be enough for the maintenance of herd protection, and the addition of a dose in infancy (1 + 1 schedule) would enhance the response to that dose and provide some protection during the first year of life. We searched PubMed from database inception to Oct 19, 2022, using search terms including but not limited to “pneumococcal conjugate vaccine”, “schedule”, “reduced-dose”, “immunogenicity”, and “carriage”. Two trials, from the UK and South Africa, have compared the immunogenicity of 1 + 1 and 2 + 1 PCV schedules (PCV13 in the UK and both PCV10 and PCV13 in South Africa). Both trials found that post-booster responses with the 1 + 1 schedules were similar to those with the 2+ 1 schedules for most serotypes. Neither trial evaluated the effects on nasopharyngeal carriage. Our previous trial in Ho Chi Minh City, Viet Nam, showed that a 1 + 1 PCV10 schedule administered at 2 months and 6 months of age was immunogenic and reduced nasopharyngeal carriage of vaccine serotypes at 18 months by 40%. We also showed that a single dose of PCV10 administered at 18 months of age was immunogenic and reduced vaccine-type carriage at 24 months by 60%. In Kenya, a 0 + 1 PCV10 schedule at 12–59 months was also shown to be immunogenic and reduced vaccine-type carriage by 47% 2 months later and by 29% 4 months later.

**Added value of this study**

To our knowledge, this is the first trial to evaluate 0 + 1 and 1 + 1 PCV schedules concurrently. The trial also incorporates a head-to-head comparison of PCV10 and PCV13 in each schedule. The primary outcome measure is vaccine-type carriage and the trial includes an unvaccinated control group, enabling estimation of the vaccine efficacy against carriage during the first 2 years of life. We show that 0 + 1 and 1 + 1 schedules of PCV10 or PCV13 all reduce carriage of vaccine serotypes during the second year of life, with a greater effect generally seen with the 1 + 1 than the 0 + 1 schedules. Such data are crucial as they provide a proxy measure for the reduction in transmission to unvaccinated individuals and therefore the probable herd protection effects of the different schedules. This trial also evaluates the immunogenicity of the different schedules, both in terms of serotype-specific IgG and functional antibody levels by opsonophagocytic assay to all serotypes in PCV10 and PCV13. A dose of PCV in infancy elicits stronger immune responses with PCV10 than PCV13, and a dose at 12 months of age elicits stronger responses with PCV13 than PCV10. With both vaccines, responses to the 12-month dose are significantly enhanced by provision of a priming dose in infancy.

**Implications of all the available evidence**

This trial adds substantially to the small body of pre-existing evidence supporting the use of reduced-dose PCV schedules. Consistent with previously published findings, data from our trial show that both 1 + 1 and 0+1 schedules of PCV10 and PCV13 reduce vaccine-type carriage and are immunogenic. With both vaccines, a 1 + 1 schedule has a greater effect on carriage and generates stronger immune responses than a 0 + 1 schedule. PCV10 is more immunogenic as a primary dose, whereas PCV13 is more immunogenic as a booster. A mixed-regimen 1 + 1 schedule with a primary dose of PCV10 and a booster dose of PCV13 warrants investigation. 1 + 1 schedules should be considered for countries with mature PCV programmes where herd protection has been established and for countries considering PCV introduction in conjunction with a comprehensive catch-up campaign. Immunogenicity and efficacy against carriage of 0 + 1 schedules support single-dose administration in humanitarian crises and remote settings where access to PCV is challenging.

## Methods

### Study design

This parallel, single-blind, randomised controlled trial was done in three districts in Ho Chi Minh City. Study clinics were established in three commune health centres, with participants recruited from those and surrounding communes. The protocol was approved by the institutional review board at the Pasteur Institute of Ho Chi Minh City. Ethical approval was obtained from the Royal Children's Hospital Melbourne human research ethics committee and the Viet Nam Ministry of Health ethics committee. The protocol has been published previously.^12^

### Participants

Infants with no clinically significant maternal or perinatal history born at 36 weeks' gestation or more were recruited by community health staff in districts 4, 7, and 8 of Ho Chi Minh City at 2 months of age and followed up to 24 months. Exclusion criteria included known allergy to any vaccine component, allergic or anaphylactic reaction to any previous vaccine, known immunodeficiency disorder, mother with a known HIV-positive status, and previous receipt of any routine 2-month vaccines. A parent or guardian provided written informed consent for each participant. Full details of the participant eligibility criteria, recruitment, and consent processes have been described previously.^12^

### Randomisation and masking

Participants were randomly assigned to one of five groups in a 4:4:4:4:9 allocation ratio. The first 200 participants per intervention group were further randomised to one of four subgroups and contribute to the immunology substudy, along with a randomly selected 200 of the first 450 participants from the control group. The randomisation was done by a database manager in Australia using a computer-generated list in a block-randomisation scheme (block size 25) stratified by city district. The group (and subgroup) allocation was contained within sealed envelopes at the study clinics, and participants were allocated by a study doctor using the next available envelope. All laboratory-based outcome assessors were masked to the group allocation but participants and those administering vaccines were not.

### Procedures

Intervention group participants received one of four PCV schedules: PCV10 at 12 months of age (0 + 1 PCV10), PCV13 at 12 months of age (0 + 1 PCV13), PCV10 at 2 months and 12 months of age (1 + 1 PCV10), or PCV13 at 2 months and 12 months of age (1 + 1 PCV13). Control group participants received a dose of PCV10 at the final study visit at 24 months of age. Additional details regarding vaccine administration have been described previously.^12^ Nasopharyngeal swabs were collected at 6 months and 12 months (for the intervention groups) and at 18 months and 24 months (all groups; appendix 2 p 2). Immunology substudy participants provided three (for the intervention groups) or one (for the control group) blood samples at selected times from 2 months, 3 months, 12 months, 13 months, and 24 months (appendix 2 p 2). Data for unvaccinated participants came from the 0 + 1 schedule groups combined, up to and including 12 months of age, and from the control group after 12 months of age. Incorrect vaccine administration and incorrect sample collection were considered protocol deviations.

Nasopharyngeal swabs were collected, stored, and tested consistent with WHO guidelines.^13^ Swabs collected at 6 months and 12 months were tested using traditional culture methods (including colonial morphology, α-haemolysis, and optochin susceptibility) with latex agglutination and Quellung serotyping.^14^ Swabs collected at 18 months and 24 months were tested using quantitative real-time PCR targeting the *lytA* gene.^15^ Samples that were *lytA* positive (or equivocal) were cultured on selective agar before molecular serotyping by microarray.^16^ Blood samples were analysed for serotype-specific IgG concentrations to all 13 serotypes in PCV13, using a modified WHO ELISA,^17^ and for functional antibody concentrations to the same serotypes using a multiplexed opsonophagocytic assay.^18^ Detailed laboratory methods have been described previously.^12^

### Outcomes

The primary outcome was the efficacy of a 0 + 1 or 1 + 1 schedule of PCV10 or PCV13 against vaccine-type pneumococcal carriage. Vaccine-type carriage was defined as carriage of any serotype in the vaccine received: PCV10-type carriage (ie, carriage of serotype 1, 4, 5, 6B, 7F, 9V, 14, 18C, 19F, or 23F) for the PCV10 groups, and PCV13-type carriage (ie, carriage of any PCV10 serotype or serotype 3, 6A, or 19A) for the PCV13 groups. The primary endpoint was 24 months, with secondary endpoints of 6 months, 12 months (for 1 + 1 groups), and 18 months (for all groups). Secondary microbiological outcomes were the comparative effect of the two vaccines (administered in the same schedule) on PCV10-type and PCV13-type carriage, and the comparative effect of the two schedules (with the same vaccine) on vaccine-type carriage. Additional microbiological outcome measures were: carriage of any pneumococcal serotype, non-PCV13-type carriage, serotype 3, 6A, or 19A carriage, serotype-specific carriage, PCV10-type carriage for the PCV13 groups, PCV13-type carriage for the PCV10 groups, and pneumococcal carriage density. Immunological outcomes were the serotype-specific immunogenicity (IgG and opsonophagocytic assay) of 0 + 1 or 1 + 1 schedules of PCV10 or PCV13 compared with unvaccinated participants, the comparative immunogenicity of PCV10 and PCV13, and the comparative immunogenicity of 0 + 1 and 1 + 1 schedules. Additional immunological outcomes were measured but will be reported separately as analyses are still underway. These outcomes were memory B cells for selected serotypes, the kinetics of the immune response to the 12-month dose of PCV, and comparing IgG and memory B-cell responses 7-days and 28-days post-vaccination. Serious adverse events were ascertained throughout the study and followed until recovery or a stable condition was reached. Adverse events that could contraindicate further vaccination were assessed following all vaccination visits. Reactogenicity was assessed following administration of each dose of PCV. Erythema at the vaccination site and axillary temperature were measured by the parent on days 0–3 post-PCV and recorded on a parent-held diary card.

### Statistical analysis

Sample size calculations were based on the reduction in vaccine-type carriage at 24 months comparing vaccinated and unvaccinated groups and the total feasible study size. The allocation ratio of 4:4:4:4:9 maximises the power for a total sample size of 2500, providing 82% power to detect a 40% reduction in vaccine-type carriage in each intervention group compared with controls, with a two-sided type I error rate of 5%. We assumed 15% vaccine-type carriage prevalence among unvaccinated participants at 24 months and 10% loss to follow-up, based on a previous trial^19^ in the same setting.

Carriage prevalence was presented as percentages with 95% CIs. The percent reduction in vaccine-type carriage, or vaccine efficacy against carriage, was calculated for each of the intervention groups compared with unvaccinated participants as (1 – prevalence ratio) × 100, both as a crude estimate and adjusted for the stratification variable district. Vaccine-type carriage prevalence was also compared between the PCV10 and PCV13 groups (in the same schedule) and between the 0 + 1 and 1 + 1 groups (with the same vaccine). Between-group comparisons of vaccine-type carriage were based on χ^2^ tests, with the two-sided p values reported. A summary measure of carriage prevalence at any time between 6 months and 24 months (for the intervention groups) and at 18 months or 24 months (for all groups) was also calculated. Carriage-density data were summarised as median log_10_ genome equivalents per mL (log_10_ GE per mL) with IQRs.

Serotype-specific IgG data were summarised as geometric mean antibody concentrations (GMCs) with 95% CIs, and the percentage of participants with protective antibody concentrations (defined as ≥0·35 μg per mL) with 95% CIs. Opsonophagocytic assay data were summarised as geometric mean opsonisation indices (GMOIs) with 95% CIs, and the percentage of participants with protective functional antibody concentrations (defined as opsonisation indices [OIs] ≥8) with 95% CIs. Between-group comparisons were based on independent *t* tests for means and on Fisher's exact tests for percentages, with values described as higher in one group if p<0·05. GMC and GMOI ratios (1 + 1 divided by 0 + 1 with 95% CIs) were calculated pre-12 month and post-12 month dose of PCV to compare the 1 + 1 and 0 + 1 schedules of PCV10 or PCV13.

Data collection, entry, and management processes have been described previously.^12^ Statistical analyses were conducted using Stata (version 15.1) or GraphPad Prism (version 9.1.1). All analyses were done on the intention-to-treat population, with all participants analysed in the group they were randomised to. Nasopharyngeal samples for which serotyping could not be done or a serotyping result could not be obtained were excluded from all analyses. Statistical analyses of secondary outcomes were not adjusted for multiplicity. Given that the per-comparison-wise error rate does not increase with multiple testing, secondary microbiological outcomes are reported in terms of specific effects, and immunological outcomes are described in terms of the patterns across serotypes.^20^ The trial was overseen by a data safety and monitoring board and is registered at ClinicalTrials.gov, NCT03098628.

### Role of the funding source

The Bill & Melinda Gates Foundation contributed to refinement of the study design. The foundation had no role in data collection, data analysis, data interpretation, or writing of the report.

## Results

**Figure 1:**
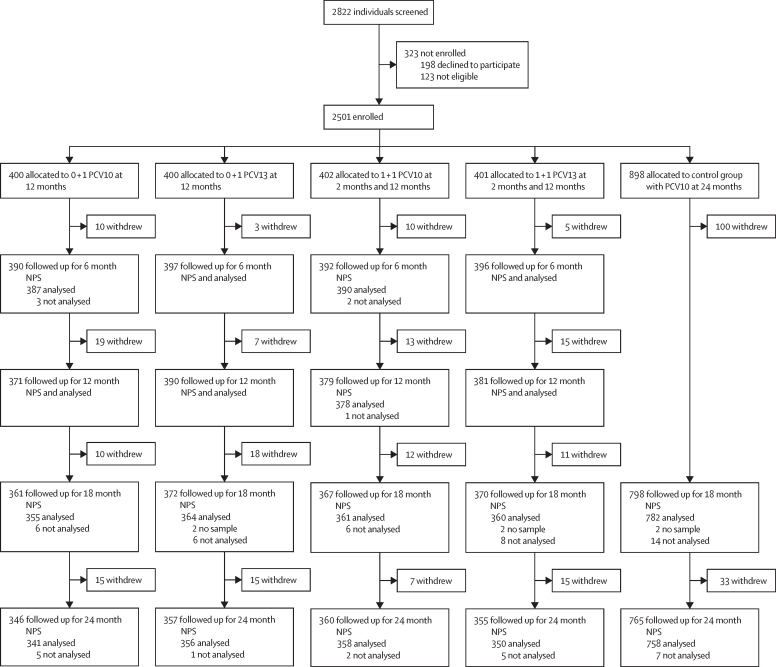
Trial profile Reasons for non-eligibility: 75 had significant medical history, 22 lived or had planned to move outside study area, 15 were outside enrolment age, and 11 had already received their routine 2-month vaccines. Reasons for withdrawal: 184 moved away and were lost to follow-up, 55 voluntarily withdrew, 44 received PCV outside the study, 20 refused study procedure, 3 died, and 12 withdrew for other reasons. Six participants did not have a sample collected at 18 months, a further 66 samples were not analysed for the following reasons: if the swabs were not handled appropriately (two 6-month swabs were inadvertently put in the same tube, one each from the two PCV10 groups), if pneumococcal carriage status could not be determined (n=2, both from the control group at 18 months), or if a serotype could not be determined (n=62). PCV=pneumococcal conjugate vaccine. PCV10=10-valent PCV. PCV13=13-valent PCV. NPS=nasopharyngeal swab.

Between March 8, 2017, and July 24, 2018, 2822 participants were screened, 2501 (88·6%) of whom were enrolled and randomly assigned to study groups: 400 to 0 + 1 PCV10, 400 to 0 + 1 PCV13, 402 to 1 + 1 PCV10, 401 to 1 + 1 PCV13, and 898 to control (figure 1). 2356 participants (94% of those enrolled) were followed up to 12 months, the time of the last PCV dose in the intervention groups, and 2183 (87%) were followed up to 24 months. The groups were balanced with respect to baseline characteristics (table 1), as were the population analysed at 24 months (appendix 2 p 3).

**Table 1:** Baseline characteristics

		**0+1 PCV10 (n=400)**	**0+1 PCV13 (n=400)**	**1+1 PCV10 (n=402)**	**1+1 PCV13 (n=401)**	**Control group (n=898)**
Sex						
	Male	207 (51·8%)	203 (50·8%)	211 (52·5%)	216 (53·9%)	467 (52·0%)
	Female	193 (48·3%)	197 (49·3%)	191 (47·5%)	185 (46·1%)	431 (48·0%)
District						
	4	149 (37·25%)	150 (37·5%)	150 (37·3%)	149 (37·2%)	337 (37·5%)
	7	105 (26·25%)	104 (26·0%)	105 (26·1%)	105 (26·2%)	234 (26·1%)
	8	146 (36·5%)	146 (36·5%)	147 (36·6%)	147 (36·7%)	327 (36·4%)
Birthweight (g)		3220 (395)	3224 (387)	3215 (402)*	3191 (380)	3217 (384)*
Place of delivery						
	Hospital	387 (96·75%)	382 (95·5%)	383 (95·3%)	386 (96·3%)	867 (96·6%)*
	Other	13 (3·3%)	18 (4·5%)	19 (4·7%)	15 (3·7%)	30 (3·3%)
Type of delivery						
	Vaginal	221 (55·3%)	240 (60·0%)	226 (56·2%)	207 (51·6%)	493 (54·9%)*
	Elective caesarean	143 (35·8%)	128 (32·0%)	135 (33·6%)	137 (34·2%)	307 (34·2%)
	Emergency caesarean	36 (9·0%)	30 (7·5%)	37 (9·2%)	54 (13·5%)	93 (10·4%)
	Other or unknown	0 (0%)	2 (<0·5%)	4 (1·0%)	3 (<0·8%)	4 (<0·5%)
Cigarette smoker in house						
	No	164 (41·0%)	149 (37·3%)	170 (42·3%)	176 (43·9%)	380 (42·3%)*
	Yes	236 (59·0%)	251 (62·8%)	232 (57·7%)	225 (56·1%)	516 (57·5%)
Breastfed						
	No	84 (21·0%)	83 (20·8%)	71 (17·7%)	88 (22·0%)	172 (19·2%)
	Yes	316 (79·0%)	317 (79·3%)	331 (82·3%)	313 (78·1%)	726 (80·9%)

Data are n (%) or mean (SD). Percentages might not sum to 100 because of rounding. PCV=pneumococcal conjugate vaccine. PCV10=10-valent PCV. PCV13=13-valent PCV.

* Missing data: delivery information (birthweight, place of delivery, and type of delivery), one participant (control group); birthweight, one other participant (1 + 1 PCV 10 group); cigarette smoker in house, two participants (control group).

**Figure 2:**
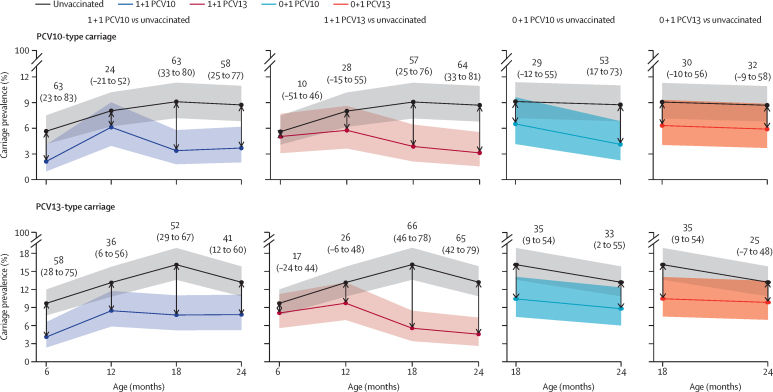
Vaccine efficacy against vaccine-type pneumococcal carriage Carriage prevalence and percent reduction in carriage of vaccine serotypes comparing each vaccine schedule with unvaccinated participants. Lines represent carriage prevalence with shaded bands showing 95% CI. Text above points shows the percent reduction (95% CI) in carriage in the vaccinated group compared with unvaccinated participants. The percent reduction is equivalent to the vaccine efficacy against carriage, calculated as (1 – prevalence ratio) × 100. At 6 months and 12 months (1 + 1 comparisons), data for unvaccinated participants came from the two 0 + 1 groups combined; at 18 and 24 months (all comparisons), data for unvaccinated participants come from the control group. PCV=pneumococcal conjugate vaccine. PCV10=10-valent PCV. PCV13=13-valent PCV.

A total of 7475 swabs were collected, of which 1290 (17·3%) contained capsular pneumococci (appendix 2 p 2). We calculated the vaccine efficacy of 1 + 1 and 0 + 1 schedules of PCV10 or PCV13 against vaccine-type carriage at 6 months and 12 months (for 1 + 1) and 18 months and 24 months (for 1 + 1 and 0 + 1; figure 2). All schedules reduced vaccine-type carriage at one or more timepoints compared with unvaccinated participants, for whom PCV10-type carriage ranged from 5·6–9·1% and PCV13-type carriage ranged from 9·7–16·1% (appendix 2 p 4). At the primary endpoint of 24 months of age, a 1 + 1 PCV10-schedule led to a 58% (95% CI 25 to 77) reduction in PCV10-type carriage (3·6% for 1 + 1 PCV10 compared with 8·7% for control) and a 1 + 1 PCV13-schedule led to a 65% (42 to 79) reduction in PCV13-type carriage (4·6% for 1 + 1 PCV13 compared with 13·2% for control). A 0 + 1 PCV10-schedule led to a 53% (17 to 73) reduction in PCV10-type carriage (4·1% for 0 + 1 PCV10 compared with 8·7% for control) and a 0 + 1 PCV13-schedule led to a non-significant 25% (–7 to 48) reduction in PCV13-type carriage (9·8% for 0 + 1 PCV13 compared with 13·2% for control). Across the other timepoints, point estimates for both PCV10-type and PCV13-type carriage prevalence in each vaccinated group were lower than for unvaccinated participants, although some of the 95% CIs for the percent reductions spanned zero (figure 2). There were no differences between the crude percent reductions and those adjusted for the stratification variable district (appendix 2 p 5).

We directly compared the vaccine-type carriage prevalence between the two vaccines (1 + 1 PCV10 *vs* 1 + 1 PCV13 and 0 + 1 PCV10 *vs* 0 + 1 PCV13) and few differences were observed. With a 1 + 1 schedule, the PCV10 group had lower carriage at 6 months of age than the PCV13 group (2·1% *vs* 5·1% for PCV10-type carriage, p=0·023; 4·1% *vs* 8·1% for PCV13-type carriage, p=0·020), with no evidence of a difference observed at the other timepoints. Considering the overall prevalence of vaccine-type carriage among participants with samples at all four timepoints (n=350 in the PCV10 groups, n=340 in the PCV13 groups) as a summary measure of the comparative risk of carriage during the first two years of life, there was no evidence of a difference between groups (appendix 2 p 6). With a 0 + 1 schedule there was no evidence of a difference in vaccine-type carriage prevalence between the PCV10 and PCV13 groups at the 18-month or 24-month timepoints, either individually or combined (appendix 2 pp 4, 6).

We directly compared the vaccine-type carriage prevalence between the two schedules (1 + 1 PCV10 *vs* 0 + 1 PCV10 and 1 + 1 PCV13 *vs* 0 + 1 PCV13) at 18 months and 24 months of age. PCV10-type carriage prevalence at 18 months was 3·3% in the 1 + 1 PCV10 group compared with 6·5% in the 0 + 1 PCV10 group (p=0·050), with a similar prevalence observed in both groups at 24 months (3·6% in the 1 + 1 group and 4·1% in the 0 + 1 group; p=0·75). PCV13-type carriage prevalence at 18 months was 5·6% in the 1 + 1 PCV13 group compared with 10·4% in the 0 + 1 PCV13 group (p=0·016), and 4·6% in the 1 + 1 PCV13 group compared with 9·8% in the 0 + 1 PCV13 group at 24 months (p=0·007).

Non-PCV13-type carriage ranged from 6·8% to 8·3% among unvaccinated participants and was generally similar across all groups (appendix 2 p 4). Serotype 3, 6A, and 19A carriage ranged from 4·1% to 7·7% among unvaccinated participants and was lowest in the 1 + 1 PCV13 group. Serotype 3 carriage was rare with only seven carriage episodes across all groups and timepoints. Serotype 6A was the serotype carried more often (appendix 2 p 7). Other commonly carried serotypes were 15A, 15B/C, 23F, 6B, 19A, and 23A, which, together with 6A, accounted for 84% of pneumococci identified. Among all pneumococcal carriers, the median carriage density was 6·2 log_10_GE per mL (IQR 5·4–6·9). Similar densities were observed across study groups and carriage outcomes (appendix 2 p 9).

**Figure 3:**
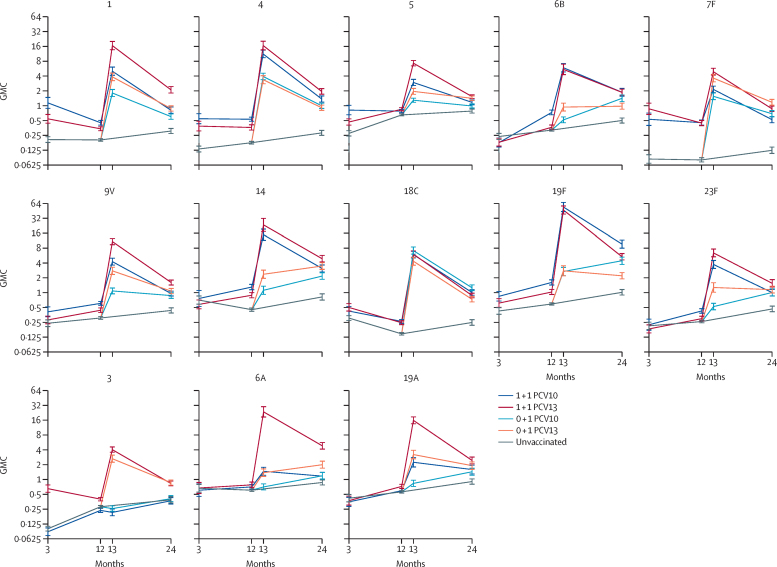
Serotype-specific GMCs (95% CI) over time Error bars indicate 95% CI. Data for unvaccinated participants comes from the two 0 + 1 groups combined at 3 months (blood sample collected at 2 months in 0 + 1 PCV10 and at 3 months in 0 + 1 PCV13) and 12 months and from the control group at 24 months. GMC=geometric mean concentration of IgG antibody. PCV=pneumococcal conjugate vaccine. PCV10=10-valent PCV. PCV13=13-valent PCV.

We assessed the immunogenicity of the different schedules among a subset of participants after 2-month PCV, before and after 12-month PCV, and at 24 months of age (appendix 2 p 10). Following a 2-month dose of either PCV10 or PCV13, vaccinated participants had higher IgG GMCs than unvaccinated participants for most serotypes both before 2-month PCV and before 12-month PCV (figure 3, appendix 2 pp 11–12). The 12-month dose in both schedules (0 + 1 and 1 + 1) with either vaccine elicited strong antibody responses to all vaccine serotypes. Elevated antibody concentrations persisted until 24 months of age, where GMCs were higher in each vaccine group than in controls for all vaccine serotypes. Directly comparing the two vaccines, after the 2-month dose PCV10 tended to generate higher GMCs than PCV13 (for four of ten serotypes after 2-month PCV and for seven of ten serotypes before 12-month PCV; appendix p 12). By contrast, after the 12-month dose PCV10 tended to generate lower GMCs than PCV13 (for seven of ten serotypes both after 12-month PCV and at 24 months of age with the 1 + 1 schedule, and for seven of ten serotypes after 12-month PCV and five of ten serotypes at 24 months of age with the 0 + 1 schedule). For the three PCV13-only serotypes, GMCs to serotype 3 were higher among PCV13 recipients than PCV10 recipients at all timepoints. GMCs to serotype 6A were similar among PCV13 recipients and PCV10 recipients at 3 months and 12 months (for the 1 + 1 schedule) and higher among PCV13-recipients at 13 months and 24 months (for 1 + 1 and 0 + 1 schedules). GMCs to serotype 19A were similar at 2 months but higher among PCV13 recipients than PCV10 recipients at 12 months (1 + 1 schedule), and at 13 months and 24 months (for the 1 + 1 and 0 + 1 schedules). GMCs were higher among PCV10 recipients than unvaccinated participants at 12 months for serotype 6A (for the 1 + 1 schedule) and at 24 months for serotypes 6A and 19A (for the 1 + 1 and 0 + 1 schedules).

We evaluated the probable degree of protection between doses afforded by a 1 + 1 schedule of PCV10 or PCV13 in terms of the percentage of participants with protective levels of IgG 4 weeks after the first dose of PCV (at 3 months of age), and of both IgG and functional antibody before the second dose of PCV (at 12 months of age). At 3 months, few unvaccinated participants had IgG of 0·35 μg per mL or more for most serotypes (appendix 2 p 13), and percentages were higher among PCV recipients than unvaccinated participants for six of ten serotypes with PCV10 and for seven of 13 serotypes with PCV13 (appendix 2 pp 13–14). At 12 months, the percentage of participants with IgG of 0·35 μg per mL or more was higher for nine of ten serotypes with PCV10 and for eight of 13 serotypes with PCV13 than among unvaccinated participants. Similarly, the percentage with OI of 8 or more was higher for ten of ten serotypes among PCV10 recipients and eight of 13 serotypes among PCV13 recipients than among unvaccinated participants, although fewer participants achieved protective concentrations of functional antibodies than IgG for most serotypes (appendix 2 p 16). Directly comparing the two vaccines, the results generally reflected the GMC data, with a higher percentage of PCV10 recipients achieving protective levels for three of ten shared serotypes at 3 months for IgG and for five of ten serotypes at 12 months for IgG and opsonophagocytic assay than for PCV13 recipients. For the three additional PCV13 serotypes, at both timepoints and by both measures, the percentages of participants with protective concentrations to serotype 3 were higher among PCV13 recipients than either PCV10 recipients or unvaccinated participants, and the percentages with protective concentrations to serotypes 6A and 19A were similar across all three groups.

We compared the immune response to a 12-month dose of PCV10 or PCV13 when given as a booster dose (for the 1 + 1 schedule) or as the first dose (for the 0 + 1 schedule). Both schedules elicited strong responses to the 12-month dose to most vaccine serotypes for both IgG and opsonophagocytic assay (figure 3, table 2, appendix 2 p 11). With PCV10, pre-12-month IgG GMCs and GMOIs were higher in the 1 + 1 group than the 0 + 1 group for ten of ten serotypes (GMC ratios 1·2–5·6, GMOI ratios 1·7–24·9; appendix 2 p 17); after 12 months, the same was true for nine of ten serotypes for IgG GMCs (GMC ratios 1·4–20·2) and seven of ten serotypes for GMOIs (GMOI ratios 3·7–36·4). With PCV13, pre-12-month IgG GMCs were higher in the 1 + 1 than the 0 + 1 group for 11 of 13 serotypes (GMC ratios 1·2–5·7) and GMOIs were higher for nine of 13 serotypes (GMOI ratios 1·7–36·0); post-12-month PCV13, the same was true for 13 of 13 serotypes for IgG GMCs (GMC ratios 1·3–17·1) and 12 of 13 serotypes for GMOIs (GMOI ratios 1·8–23·1). At 24 months of age, GMCs were still higher in the 1 + 1 than the 0 + 1 groups for five of ten serotypes with PCV10 and for eight of 13 serotypes with PCV13.

**Table 2:** Geometric mean opsonisation index pre-4-weeks and 4-weeks post-12-month dose of PCV

	**Pre-12-month PCV**			**Post-12-month PCV**			
	0+1 PCV10*	1+1 PCV10	1+1 PCV13	0+1 PCV10	0+1 PCV13	1+1 PCV10	1+1 PCV13
**PCV10 serotypes**							
1	4 (4–5)	8 (6–10)	5 (4–6)	26 (17–40)	74 (44–123)	350 (224–548)	840 (575–1227)
4	4 (4–5)	8 (6–11)	8 (5–13)	463 (245–877)	1439 (936–2213)	2246 (1641–3075)	5140 (3952–6687)
5	4 (4–4)	15 (11–22)	11 (8–16)	21 (16–29)	73 (44–122)	782 (576–1061)	1688 (1250–2280)
6B	6 (4–9)	18 (10–31)	4 (4–5)	42 (19–92)	414 (179–955)	1519 (1101–2095)	3889 (2267–6672)
7F	9 (5–15)	219 (126–381)	317 (179–561)	1605 (1008–2557)	4227 (2739–6524)	2340 (1718–3189)	6534 (5354–7974)
9V	6 (4–8)	49 (28–86)	17 (9–32)	813 (426–1549)	3732 (2455–5675)	3000 (2283–3941)	9582 (7612–12 062)
14	28 (13–62)	160 (75–345)	173 (82–366)	3016 (1851–4914)	6672 (4389–10 141)	4176 (2729–6389)	15 081 (11 147–20 403)
18C	4 (4–4)	9 (7–13)	8 (6–11)	3889 (2422–6243)	1915 (1220–3005)	6194 (4489–8548)	3465 (2735–4390)
19F	5 (4–6)	65 (35–118)	13 (9–19)	1032 (579–1837)	800 (443–1447)	6120 (4678–8006)	6192 (4802–7986)
23F	6 (4–9)	13 (8–21)	9 (5–16)	133 (58–305)	1250 (610–2562)	1049 (636–1731)	5884 (4213–8217)
**Additional PCV13 serotypes**							
3	8 (6–10)	8 (6–10)	23 (16–35)	8 (6–11)	357 (250–510)	8 (6–12)	815 (628–1057)
6A	6 (4–9)	9 (5–14)	8 (5–13)	53 (22–125)	1809 (952–3438)	171 (76–388)	9540 (7002–12 998)
19A	6 (5–7)	9 (6–13)	9 (7–13)	93 (59–145)	663 (440–1001)	215 (128–361)	3167 (2332–4301)

Data are geometric means (95% CI). PCV=pneumococcal conjugate vaccine. PCV10=10-valent PCV. PCV13=13-valent PCV.

* Pre-12-month PCV opsonophagocytic assays were only done in one of the two 0 + 1 groups, as these groups have the same vaccination status up to 12 months of age.

There were 415 serious adverse events resulting in hospital admissions among 337 participants over the course of the study (appendix 2 p 18). Most were unrelated to vaccination (n=411, 99·0%), and the most common reasons for admission were respiratory illness (n=185, 44·6%), diarrhoea or vomiting (n=52, 12·5%), fever (n=44, 10·6%), ear-nose-and-throat conditions (n=38, 9·2%), and hand-foot-and-mouth disease (n=37, 8·9%). Most serious adverse events resolved without sequelae (n=412, 99·3%). There were three deaths, one from an accident (participant aged 3 months from the control group), one from a congenital heart condition (participant aged 4 months from the 0 + 1 PCV10 group), and one from *Pseudomonas* pneumonia (participant aged 24 months from the control group). Diary cards were collected following 2271 (97·8%) of 2322 PCV doses (appendix 2 p 18), with reactions reported in 319 (13·7%) cards. Reactions were more commonly reported following the 2-month dose than the 12-month dose but did not differ between groups at either timepoint.

## Discussion

Interest in reduced-dose schedules is growing as a more efficient way to administer PCV where vaccine-type disease has been controlled and herd protection becomes the primary aim of vaccination. Herd protection is mediated through reduction in nasopharyngeal carriage of vaccine serotypes leading to reduced pneumococcal transmission. The effect of vaccination on carriage therefore offers a good proxy measure for assessing herd protection, yet data on the effect of reduced-dose schedules on carriage are scarce. A dose in the second year of life should be sufficient to maintain herd protection,^4^ while a dose in infancy is likely to offer some degree of individual protection during the first year of life and provides immunological priming for the second dose. This trial provides a head-to-head evaluation of the effect on carriage and the immunogenicity of PCV10 and PCV13 in a 1 + 1 schedule (at 2 months and 12 months of age) and a 0 + 1 schedule (a single dose at 12 months of age). The inclusion of an unvaccinated control group enabled us to calculate the vaccine efficacy of each schedule on carriage, in addition to comparing the different schedules and vaccine formulations.

At 24 months of age, the vaccine efficacy of a 1 + 1 PCV10 schedule against PCV10-type carriage was 58% and the vaccine efficacy of a 1 + 1 PCV13 schedule against PCV13-type carriage was 65%. Similar efficacies were observed at 18 months of age (63% for 1 + 1 PCV10 and 66% for 1 + 1 PCV13). These results represent similar or greater reductions than observed following a 2 + 1 schedule of PCV10 or PCV13 in an earlier trial from the same setting.^19^ Both 1 + 1 schedules therefore show a strong effect on vaccine-type carriage during the second year of life that should be sufficient to generate and maintain herd protection. The 0 + 1 PCV10 schedule had a vaccine efficacy of 53% against PCV10-type carriage at 24 months. This reduction is only slightly lower than the reduction seen with the 1 + 1 PCV10 schedule and is also in line with the 60% reduction observed following a single dose of PCV10 administered at 18 months of age in the earlier trial in Ho Chi Minh city.^11^ The 0 + 1 PCV13 schedule had a non-significant vaccine efficacy of 25% against PCV13-type carriage at 24 months, but both 0 + 1 schedules had vaccine efficacies of 35% against PCV13-type carriage at 18 months. Overall, the 0 + 1 schedules tend to have a lower effect on carriage than the 1 + 1 schedules, but still lead to substantial reductions in vaccine-type carriage. Such a schedule is particularly useful when provision of multiple doses of PCV is not feasible, such as in remote settings and during humanitarian crises.

We evaluated the immunogenicity of the different schedules by ELISA and opsonophagocytic assay to establish the probable protection during the first year of life afforded by a 2-month dose of PCV, the priming effect of an infant dose of PCV on responses to a 12-month dose, and how the responses to PCV10 and PCV13 compare. The first two relate to the added advantages that a 1 + 1 schedule offers over a 0 + 1 schedule. Low-income and middle-income countries (LMICs) tend to have higher residual vaccine-type carriage following PCV introduction than high-income countries,^21^ leaving children at greater risk of pneumococcal disease. When considering a switch to a reduced-dose schedule, the probable protection offered by the first dose in a 1 + 1 schedule is an important factor. Here, we show a reasonable response to a 2-month dose of PCV10 or PCV13 for most serotypes, consistent with the 1 + 1 trials from the UK and South Africa and with the post-dose-one immunogenicity data from the previous trial in Viet Nam.^6^, ^7^, ^22^ As with these other studies, the notable exceptions were serotypes 6B and 23F, with no response 4 weeks after either vaccine. Serotypes 6B and 23F are known to be poorly immunogenic but might require lower antibody levels for protection, somewhat reducing this concern.^23^, ^24^ Overall, results from this and previous studies suggest that a single dose of PCV in infancy is likely to provide some degree of individual protection against pneumococcal disease. Comparison of the 1 + 1 and 0 + 1 schedules show that this single dose in infancy also has a strong priming effect, with higher post-12-month IgG and opsonophagocytic assay levels in the 1 + 1 than 0 + 1 groups for most serotypes. Although not as profound as with the 1 + 1 schedules, good responses were still observed with the 0 + 1 schedules. Notably, at 24 months of age, IgG concentrations for all vaccine serotypes were higher in each of the four vaccine groups than in unvaccinated participants. This finding shows that a 12-month dose of PCV is immunogenic, and responses are substantially enhanced by provision of a priming dose in infancy.

The head-to-head product comparison shows that immune responses to a 2-month dose, both 4 weeks after PCV (by ELISA) and at 12 months of age (by ELISA and opsonophagocytic assay), tend to be better with PCV10 than PCV13, including at 12 months for the problematic serotypes 6B and 23F. These results are consistent with previous post-dose-one head-to-head data from Viet Nam,^22^ Australia,^25^ and South Africa,^7^ and is supported by our carriage finding that PCV10 reduced vaccine-type carriage at 6 months of age but PCV13 did not. Conversely, immune responses to a 12-month dose, whether administered as a first-dose or a booster-dose, tend to be better with PCV13 than PCV10, consistent with the South African trial^7^ (on the basis of non-overlapping 95% CIs as this trial did not report any head-to-head comparisons). Despite these differences, both vaccines had a substantial effect on carriage in the second year of life, including reductions in PCV13-type carriage following PCV10 vaccination that suggest some degree of cross protection of serotype 6A and 19A carriage with PCV10. Of the seven serotypes for which PCV13 produced higher post-12-month-dose IgG GMCs than PCV10, six had higher GMCs with PCV10 than PCV13 either post-2-month dose or pre-12-month dose. These serotypes include serotypes 1 and 5, which are of particular importance in LMICs. These results suggest that a mixed regimen, consisting of an infant-dose of PCV10 and a booster-dose of PCV13, could offer a strategy to maximise the effectiveness of reduced-dose schedules and should be evaluated in a future study. Various mixed-regimen PCV schedules involving two-dose or three-dose primary series have previously been shown to be safe and immunogenic,^25^, ^26^, ^27^, ^28^, ^29^ but our finding of stronger immunogenicity of a one-dose primary series with PCV10 and stronger immunogenicity of a booster dose with PCV13 makes this approach particularly interesting in the context of reduced-dose schedules.

One of the strengths of this study is the inclusion of both PCV10 and PCV13 administered in two different schedules along with an unvaccinated control group. However, this strength results in multiple comparisons between groups. To overcome the multiple comparisons we have reported precise and specific microbiological outcomes, recognising that the per-comparison-wise error rate does not increase with multiple testing so adjusting for multiplicity is not necessary.^20^ Similarly, immunological assessments are made on multiple serotypes at each timepoint, which is a problem faced by all studies of PCVs, so we have described the immunological outcomes in terms of the patterns across serotypes. Vaccine-type carriage prevalence was lower than anticipated and loss to follow-up was higher than anticipated, increasing the potential for type II error. Loss to follow-up also led to missing outcome data, although baseline characteristics were balanced across groups among those analysed, so the potential for bias is minimal. A limitation of this study is the use of different serotyping methods at the 6-month and 12-month timepoints (latex agglutination and Quellung) and the 18-month and 24-month timepoints (microarray). These differences are mitigated by the fact that all comparisons are made between groups at the same timepoint, both methods involve a culture step so are likely to have similar sensitivity in detecting pneumococci, and multiple serotype carriage in this study was low.

In conclusion, a 1 + 1 schedule of PCV10 or PCV13 at 2 months and 12 months substantially reduces vaccine-type pneumococcal carriage during the second year of life and elicits strong immune responses. Such a schedule is likely to generate substantial herd protection along with some individual protection during the first year of life and is suitable for mature PCV programmes or for introduction in conjunction with an extensive catch-up campaign. Potentially the most effective way to deliver a 1 + 1 schedule could be a primary dose of PCV10 with a booster dose of PCV13; this regimen warrants further investigation. Reduced-dose schedules with the recently developed 10-valent PCV Pneumosil (Serum Institute of India, Pune), which has been shown to be immunologically non-inferior to PCV10 in a 3 + 0 schedule,^30^ should also be evaluated. A 0 + 1 schedule of PCV10 or PCV13 at 12 months also has some effect on vaccine-type pneumococcal carriage and is immunogenic. Such a schedule could be considered for use in difficult circumstances such as during humanitarian crises and in remote settings, to offer some protection to vulnerable populations.

## Data sharing

The study protocol and informed consent form have been published previously and are freely available. Data will be made publicly available in accordance with the rules set out by the Bill & Melinda Gates Foundation. Requests can be made by contacting the corresponding author.

## Declaration of interests

All authors received salary support from the Bill & Melinda Gates Foundation grant. KM and CS are investigators on a clinical research collaboration with Pfizer on PCV vaccination in Mongolia and are investigators on a Merck Investigator Studies Program grant funded by MSD on pneumococcal serotype epidemiology in children.

## Supplementary Materials

Vietnamese translation of the abstract
