# Supplementary material for: Efficacy against pneumococcal carriage and the immunogenicity of reduced-dose (0 + 1 and 1 + 1) PCV10 and PCV13 schedules in Ho Chi Minh City, Viet Nam: a parallel, single-blind, randomised controlled trial
Source: Lancet Infect Dis. 2023 Aug;23(8):933–44. doi: 10.1016/S1473-3099(23)00061-0 (PMC10371874; doi:10.1016/S1473-3099(23)00061-0)
Supplement: Vietnamese translation of the abstract [file mmc1.pdf]

THE LANCET
Infectious Diseases

Supplementary appendix 1

This translation in Vietnamese was submitted by the authors and we reproduce it as supplied. It has not been peer reviewed. The Lancet's editorial processes have only been applied to the original in English, which should serve as reference for this manuscript.

Bản dịch sang tiếng Việt này do các tác giả gửi và chúng tôi sao chép lại như đã cung cấp. Bản dịch này không qua phản biện. Quy trình biên tập của The Lancet chỉ áp dụng cho bản gốc bằng tiếng Anh, và độc giả chỉ nên dùng bản gốc tiếng Anh làm tài liệu tham khảo.

Supplement to: Temple B, Tran HP, Dai VTT, et al. Efficacy against pneumococcal carriage and the immunogenicity of reduced-dose (0 + 1 and 1 + 1) PCV10 and PCV13 schedules in Ho Chi Minh City, Viet Nam: a parallel, single-blind, randomised controlled trial. *Lancet Infect Dis* 2023; published online April 13. https://doi.org/10.1016/S1473-3099(23)00061-0.

Hiệu quả ngăn ngừa sự mang phế cầu khuẩn và khả năng sinh miễn dịch của các phác đồ giảm liều (0 + 1 và 1 + 1) của PCV10 và PCV13 tại Thành phố Hồ Chí Minh, Việt Nam: một thử nghiệm ngẫu nhiên có đối chứng mù đơn, song song

Beth Temple*, Hau Phuc Tran*, Vo Thi Trang Dai*, Heidi Smith-Vaughan*, The VPT-II Collaborator Group‡, Paul Vincent Licciardi†, Catherine Satzke†, Thuong Vu Nguyen†, Kim Mulholland†

Tóm tắt

Bối cảnh: Sự quan tâm đến các liệu trình giảm liều của vắc-xin cộng hợp ngừa phế cầu khuẩn (PCV) đang gia tăng, nhưng dữ liệu về khả năng cung cấp sự bảo vệ trực tiếp và gián tiếp của chúng còn khan hiếm. Chúng tôi đã đánh giá liệu trình 1+1 (lúc 2 tháng và 12 tháng) và 0+1 (lúc 12 tháng) của PCV10 hoặc PCV13 trong quần thể mà phần lớn chưa được tiêm phòng.

Phương pháp: Trong thử nghiệm đối chứng ngẫu nhiên, mù đơn, song song này, các trẻ sơ sinh khỏe mạnh 2 tháng tuổi được tuyển chọn từ hồ sơ khai sinh tại ba quận ở Thành phố Hồ Chí Minh, Việt Nam (4:4:4:4:9) và được phân vào một trong năm nhóm: tiêm PCV10 lúc 12 tháng tuổi (0+1 PCV10), PCV13 lúc 12 tháng tuổi (0+1 PCV13), PCV10 lúc 2 tháng và 12 tháng tuổi (1+1 PCV10), PCV13 lúc 2 tháng và 12 tháng tuổi (1 + 1 PCV13) và nhóm đối chứng không tiêm vắc xin phế cầu. Các chuyên viên đánh giá kết quả không được biết sự phân bổ nhóm (mù đơn), còn những người chăm sóc trẻ và chuyên viên tiêm vắc-xin thì biết sự phân bổ này. Các mẫu phết mũi hầu thu thập lúc 6 tháng, 12 tháng, 18 tháng và 24 tháng được phân tích để tìm phế cầu khuẩn. Các mẫu máu được thu thập từ một nhóm nhỏ những người tham gia (200 trẻ mỗi nhóm) tại các thời điểm khác nhau được phân tích bằng ELISA và xét nghiệm opsonophagocytic. Kết quả chính là hiệu quả của từng liệu trình đối với việc ngăn ngừa sự mang phế cầu týp vắc xin ở thời điểm 24 tháng, được phân tích theo ý định điều trị (intention to treat) cho tất cả những mẫu phết mũi hầu thu thập được. Thử nghiệm này được đăng ký tại ClinicalTrials.gov, NCT03098628.

Kết quả: 2501 trẻ sơ sinh được thu tuyển từ ngày 8 tháng 3 năm 2017 đến ngày 24 tháng 7 năm 2018 và được phân ngẫu nhiên vào các nhóm nghiên cứu (400 cho liệu trình 0 + 1 PCV10, 400 cho liệu trình 0 + 1 PCV13, 402 cho liệu trình 1 + 1 PCV10, 401 cho liệu trình 1 + 1 PCV13 , và 898 là nhóm chứng). Phân tích điểm cuối chính bao gồm 341 trẻ tham gia liệu trình 0 + 1 PCV10, 356 trẻ tham gia liệu trình 0 + 1 PCV13, 358 trẻ tham gia liệu trình 1 + 1 PCV10, 350 trẻ tham gia liệu trình 1 + 1 PCV13 và 758 trẻ nhóm chứng. Ở thời điểm 24 tháng, liệu trình 1 + 1 PCV10 làm giảm 58% sự mang phế cầu týp vắc xin PCV10 (KTC 95% 25 - 77), liệu trình 1 + 1 PCV13 làm giảm 65% sự mang phế cầu týp vắc xin PCV13 (KTC 95% 42 - 79), Liệu trình 0 + 1 PCV10 làm giảm 53% sự mang phế cầu týp vắc xin PCV13 (KTC 95% 17 - 73) và liệu trình 0 + 1 PCV13 giảm sự mang phế cầu týp vắc xin PCV13 không đáng kể (25% KTC–7 đến 48) so với nhóm đối chứng không được tiêm vắc xin phế cầu. Khả năng gây phản ứng và các biến cố bất lợi nghiêm trọng là tương tự nhau giữa các nhóm.

Phiên giải kết quả: Liệu trình 1 + 1 PCV làm giảm đáng kể sự mang phế cầu týp vắc-xin và có khả năng tạo ra sự bảo vệ cộng đồng đáng kể và phần nào bảo vệ cá nhân trong năm đầu đời. Liệu trình như vậy phù hợp với các chương trình tiêm vắc xin phế cầu (PCV) đã chín mùi hoặc để đưa vào kết hợp với một chiến dịch tiêm vét toàn diện và có khả năng là hiệu quả nhất khi được ứng dụng dưới dạng phác đồ hỗn hợp (PCV10 rồi đến PCV13). Liệu trình 0 + 1 PCV có một số tác động trên sự mang phế cầu khuẩn vùng mũi hầu với đáp ứng miễn dịch vừa phải và có thể được xem xét sử dụng trong các cuộc khủng hoảng nhân đạo hoặc cho các vùng sâu vùng xa.

Funding: Quỹ Bill & Melinda Gates

Bản quyền © thuộc các Tác giả (s). Được xuất bản bởi Elsevier Ltd. Đây là một bài báo Truy cập Mở theo giấy phép CC BY 4.0.
