# Supplementary material for: Efficacy against pneumococcal carriage and the immunogenicity of reduced-dose (0 + 1 and 1 + 1) PCV10 and PCV13 schedules in Ho Chi Minh City, Viet Nam: a parallel, single-blind, randomised controlled trial
Source: Lancet Infect Dis. 2023 Aug;23(8):933–44. doi: 10.1016/S1473-3099(23)00061-0 (PMC10371874; doi:10.1016/S1473-3099(23)00061-0)
Supplement: Supplementary appendix 2 [file mmc2.pdf]

THE LANCET
Infectious Diseases

Supplementary appendix 2

This appendix formed part of the original submission and has been peer reviewed. We post it as supplied by the authors.

Supplement to: Temple B, Tran HP, Dai VTT, et al. Efficacy against pneumococcal carriage and the immunogenicity of reduced-dose (0 + 1 and 1 + 1) PCV10 and PCV13 schedules in Ho Chi Minh City, Viet Nam: a parallel, single-blind, randomised controlled trial. *Lancet Infect Dis* 2023; published online April 13. https://doi.org/10.1016/S1473-3099(23)00061-0.

APPENDIX

TABLE OF CONTENTS

Table S1: Schedule of PCV doses and NP swabs 2

Table S2: Schedule of blood samples in the immunology sub-study 2

Box S1: Summary of overall microbiology findings 2

Table S3: Baseline characteristics by study group, restricted to population analysed at 24 months 3

Table S4: Pneumococcal carriage prevalence over time 4

Table S5: Percent reduction (95% CI) in vaccine-type carriage, crude and adjusted for district 5

Table S6: Overall risk of pneumococcal carriage 6

Table S7: Serotype-specific carriage prevalence over time 7

Figure S1: Pneumococcal carriage density 9

Table S8: Number of blood samples analysed for IgG and OPA 10

Table S9: Geometric Mean Concentration (95% CI) of serotype-specific IgG over time 11

Table S10: Ratio of GMCs (95% CI) comparing vaccinated and unvaccinated participants 12

Table S11: Ratio of GMCs (95% CI) comparing PCV10- and PCV13-vaccinated participants 12

Table S12: Percentage of participants (95% CI) with serotype-specific IgG ≥0.35μg/ml over time 13

Table S13: Difference (95% CI) in percentage of participants achieving serotype-specific IgG ≥0.35μg/ml and OI ≥8 after a 2-month dose of PCV, comparing vaccinated and unvaccinated participants, and comparing PCV10- and PCV13-recipients 14

Figure S2: Protection between doses afforded by a 1+1 schedule of PCV10 or PCV13 15

Table S14: Percentage (95% CI) of participants with opsonisation index (OI) ≥8 pre- and 4-weeks post-12m dose of PCV 16

Table S15: Ratio (95% CI) of geometric mean concentrations of IgG and functional antibody comparing participants who received a 1+1 or 0+1 schedule of a) PCV10 and b) PCV13 17

Table S16: Serious Adverse Events 18

Table S17: Post-PCV reactogenicity 18

Table S1: Schedule of PCV doses and NP swabs

	Group	n (target)	Schedule	Vaccine	2m	6m	12m	18m	24m
**PCV doses**	V	400	0+1	PCV10			X		
	W	400	0+1	PCV13			X		
	X	400	1+1	PCV10	X		X		
	Y	400	1+1	PCV13	X		X		
	Z	900	Controls	PCV10					X
**NP swabs**						X*	X*	X	X

* 6 and 12m swabs collected from Groups V-Y only. Swabs from Groups V and W combined (the two 0+1 groups) provide unvaccinated (control) data at these timepoints. PCV = pneumococcal conjugate vaccine. PCV10 = ten-valent PCV. PCV13 = 13-valent PCV. NP swab = nasopharyngeal swab. m = months.

Table S2: Schedule of blood samples in the immunology sub-study

Group	Subgroup	n (target)	2/3m*	Pre-12m PCV	Post-12m PCV		24m
				12m	12m+7d	12m+28d	
V-Y	a	50		X	X		X
	b	50	X	X	X		
	c	50		X		X	X
	d	50		X (OPA)†		X (OPA)	X
Z		200					X

* Sample collected at 2 months in group V and at 3 months in groups X-Y. † Pre-12m PCV sample in Group W-d not analysed by OPA. PCV = pneumococcal conjugate vaccine. m = months. OPA = opsonophagocytic assay.

Box S1: Summary of overall microbiology findings

Across all timepoints and groups a total of 7475 swabs were collected, of which capsular pneumococci was identified in 1290 (17.3%). Among unvaccinated controls, capsular pneumococcal carriage increased steadily from 14.5% at 6 months of age to a peak of 22.4% at 18 months of age before declining slightly to 20.1% at 24 months of age (Appendix Table S1). In all, 34 different serotypes were identified. A single serotype was identified from most swabs across all groups (1253/1290, 97.1%); two serotypes were identified in 36 swabs and three serotypes identified in one swab. Non-encapsulated pneumococci were identified in 285 swabs (3.8%), with four different genetic lineages detected (NT2, NT3b, NT4a, and NT4b; lineages determined at 18 and 24 months only).

Table S3: Baseline characteristics by study group, restricted to population analysed at 24 months

	0+ 1 PCV10 (Group V) (n=341)	0+1 PCV13 (Group W) (n=356)	1+1 PCV10 (Group X) (n=358)	1+1 PCV13 (Group Y) (n=350)	No PCV (Group Z) (n=758)	p-value
Sex						0.78
Male	174 (51)	177 (50)	189 (53)	190 (54)	390 (51)	
Female	167 (49)	179 (50)	169 (47)	160 (46)	368 (49)	
District						>0.999
4	137 (40)	139 (39)	141 (39)	137 (39)	299 (39)	
7	84 (25)	93 (26)	93 (26)	87 (25)	202 (27)	
8	120 (35)	124 (35)	124 (35)	126 (36)	257 (34)	
Birthweight (g)	3228 (399)	3229 (381)	3201 (398)*	3201 (383)	3223 (381)*	0.72
Place of delivery						0.61
Hospital	331 (97)	338 (95)	342 (96)	335 (96)	730 (96)*	
Other	10 (3)	18 (5)	16 (5)	15 (4)	27 (4)	
Type of delivery						0.12
Vaginal	185 (54)	213 (60)	201 (56)	174 (50)	407 (54)*	
Elective caesarean	125 (37)	112 (32)	119 (33)	123 (35)	266 (35)	
Emergency caesarean	31 (9)	29 (8)	34 (10)	50 (14)	81 (11)	
Other/unknown	0 (0)	2 (1)	4 (1)	3 (1)	3 (0)	
Cigarette smoker in house						0.67
No	145 (43)	135 (38)	151 (42)	150 (43)	315 (42)*	
Yes	196 (58)	221 (62)	207 (58)	200 (57)	442 (58)	
Breastfeeding						0.41
No	68 (20)	67 (19)	60 (17)	77 (22)	136 (18)	
Yes	273 (80)	289 (81)	298 (83)	273 (78)	622 (82)	

Data are n (%) or mean (SD). * data missing for 1 participant.

Table S4: Pneumococcal carriage prevalence over time

	0+1 PCV10 (Group V)		0+1 PCV13 (Group W)		1+1 PCV10 (Group X)		1+1 PCV13 (Group Y)		Controls*	
	**n/N**	**Prev. (95% CI)**	**n/N**	**Prev. (95% CI)**	**n/N**	**Prev. (95% CI)**	**n/N**	**Prev. (95% CI)**	**n/N**	**Prev. (95% CI)**
**Any pneumococcal serotype carriage**										
6m	59/387	15·2 (11·8-19·2)	55/397	13·9 (10·6-17·6)	42/390	10·8 (7·9-14·3)	52/396	13·1 (10·0-16·9)	114/784	14·5 (12·1-17·2)
12m	79/371	21·3 (17·2-25·8)	71/390	18·2 (14·5-22·4)	68/378	18·0 (14·2-22·2)	62/381	16·3 (12·7-20·4)	150/761	19·7 (16·9-22·7)
18m	66/355	18·6 (14·7-23·0)	73/364	20·1 (16·1-24·5)	62/361	17·2 (13·4-21·5)	46/360	12·8 (9·5-16·7)	175/782	22·4 (19·5-25·5)
24m	60/341	17·6 (13·7-22·1)	67/356	18·8 (14·9-23·3)	59/358	16·5 (12·8-20·7)	42/350	12·0 (8·8-15·9)	152/758	20·1 (17·3-23·1)
**PCV10-type carriage**										
6m	25/387	6·5 (4·2-9·4)	19/397	4·8 (2·9-7·4)	8/390	2·1 (0·9-4·0)	20/396	5·1 (3·1-7·7)	44/784	5·6 (4·1-7·5)
12m	31/371	8·4 (5·7-11·7)	30/390	7·7 (5·2-10·8)	23/378	6·1 (3·9-9·0)	22/381	5·8 (3·7-8·6)	61/761	8·0 (6·2-10·2)
18m	23/355	6·5 (4·2-9·6)	23/364	6·3 (4·0-9·3)	12/361	3·3 (1·7-5·7)	14/360	3·9 (2·1-6·4)	71/782	9·1 (7·2-11·3)
24m	14/341	4·1 (2·3-6·8)	21/356	5·9 (3·7-8·9)	13/358	3·6 (1·9-6·1)	11/350	3·1 (1·6-5·6)	66/758	8·7 (6·8-10·9)
**PCV13-type carriage**										
6m	41/387	10·6 (7·7-14·1)	35/397	8·8 (6·2-12·0)	16/390	4·1 (2·4-6·6)	32/396	8·1 (5·6-11·2)	76/784	9·7 (7·7-12·0)
12m	50/371	13·5 (10·2-17·4)	50/390	12·8 (9·7-16·6)	32/378	8·5 (5·9-11·7)	37/381	9·7 (6·9-13·1)	100/761	13·1 (10·8-15·7)
18m	37/355	10·4 (7·4-14·1)	38/364	10·4 (7·5-14·0)	28/361	7·8 (5·2-11·0)	20/360	5·6 (3·4-8·4)	126/782	16·1 (13·6-18·9)
24m	30/341	8·8 (6·0-12·3)	35/356	9·8 (6·9-13·4)	28/358	7·8 (5·3-11·1)	16/350	4·6 (2·6-7·3)	100/758	13·2 (10·9-15·8)
**Serotype 3/6A/19A carriage**										
6m	16/387	4·1 (2·4-6·6)	16/397	4·0 (2·3-6·5)	8/390	2·1 (0·9-4·0)	12/396	3·0 (1·6-5·2)	32/784	4·1 (2·8-5·7)
12m	19/371	5·1 (3·1-7·9)	21/390	5·4 (3·4-8·1)	9/378	2·4 (1·1-4·5)	15/381	3·9 (2·2-6·4)	40/761	5·3 (3·8-7·1)
18m	15/355	4·2 (2·4-6·9)	16/364	4·4 (2·5-7·0)	16/361	4·4 (2·6-7·1)	6/360	1·7 (0·6-3·6)	60/782	7·7 (5·9-9·8)
24m	16/341	4·7 (2·7-7·5)	14/356	3·9 (2·2-6·5)	15/358	4·2 (2·4-6·8)	5/350	1·4 (0·5-3·3)	37/758	4·9 (3·5-6·7)
**Non-PCV13-type carriage**										
6m	18/387	4·7 (2·8-7·3)	21/397	5·3 (3·3-8·0)	26/390	6·7 (4·4-9·6)	20/396	5·1 (3·1-7·7)	39/784	5·0 (3·6-6·7)
12m	29/371	7·8 (5·3-11·0)	21/390	5·4 (3·4-8·1)	36/378	9·5 (6·8-12·9)	25/381	6·6 (4·3-9·5)	50/761	6·6 (4·9-8·6)
18m	30/355	8·5 (5·8-11·8)	37/364	10·2 (7·3-13·7)	35/361	9·7 (6·8-13·2)	26/360	7·2 (4·8-10·4)	53/782	6·8 (5·1-8·8)
24m	31/341	9·1 (6·3-12·7)	36/356	10·1 (7·2-13·7)	31/358	8·7 (6·0-12·1)	26/350	7·4 (4·9-10·7)	55/758	7·3 (5·5-9·3)

Prev. = prevalence, expressed as a percentage. * Control data sourced from combined Group V and W data at 6 and 12 months, and from Group Z at 18 and 24 months. PCV = pneumococcal conjugate vaccine. PCV10 = ten-valent PCV. PCV13 = 13-valent PCV. CI = confidence interval.

Table S5: Percent reduction (95% CI) in vaccine-type carriage, crude and adjusted for district

	Crude percent reduction (95% CI)				Adjusted percent reduction (95% CI)			
	**6m**	**12m**	**18m**	**24m**	**6m**	**12m**	**18m**	**24m**
**1+1 PCV10**								
PCV10-type carriage	63 (23, 83)	24 (-21, 52)	63 (33, 80)	58 (25, 77)	64 (23, 83)	24 (-20, 52)	63 (23, 80)	58 (26, 77)
PCV13-type carriage	58 (28, 75)	36 (6, 56)	52 (29, 67)	41 (12, 60)	58 (28, 75)	36 (6, 56)	52 (29, 67)	41 (12, 60)
**1+1 PCV13**								
PCV10-type carriage	10 (-51, 46)	28 (-15, 55)	57 (25, 76)	64 (33, 81)	10 (-51, 46)	28 (-15, 55)	57 (25, 76)	64 (23, 81)
PCV13-type carriage	17 (-24, 44)	26 (-6, 48)	66 (46, 78)	65 (42, 79)	17 (-24, 44)	26 (-5, 48)	66 (46, 78)	66 (43, 79)
**0+1 PCV10**								
PCV10-type carriage	··	··	29 (-12, 55)	53 (17, 73)	··	··	28 (-12, 54)	53 (18, 73)
PCV13-type carriage	··	··	35 (9, 54)	33 (2, 55)	··	··	35 (9, 54)	33 (2, 55)
**0+1 PCV13**								
PCV10-type carriage	··	··	30 (-10, 56)	32 (-9, 58)	··	··	30 (-10, 55)	32 (-8, 58)
PCV13-type carriage	··	··	35 (9, 54)	25 (-7, 48)	··	··	35 (9, 54)	26 (-7, 48)

Percent reductions in vaccine-type carriage, calculated as 1 minus the prevalence ratio (vaccinated / unvaccinated) and expressed as percentages with 95% CIs. Data for unvaccinated participants come from the two 0+1 groups combined (Groups V and W) at 6 and 12 months, and from the control group (Group Z) at 18 and 24 months. PCV = pneumococcal conjugate vaccine. PCV10 = 10-valent PCV. PCV13 = 13-valent PCV. CI = confidence interval.

Table S6: Overall risk of pneumococcal carriage

Prevalence of carriage at any timepoint among participants with a complete set of samples: a) intervention group participants with swabs at 6, 12, 18, and 24 months of age; and b) intervention and control group participants with swabs at 18 and 24 months of age.

	0+1 PCV10 (Group V)		0+1 PCV13 (Group W)		1+1 PCV10 (Group X)		1+1 PCV13 (Group Y)		Controls*	
	n/N	Prev. (95% CI)	n/N	Prev. (95% CI)	n/N	Prev. (95% CI)	n/N	Prev. (95% CI)	n/N	Prev. (95% CI)
**a) Prevalence of carriage at any timepoint between 6 and 24 months of age**										
Any pneumococcal serotype carriage	150/332	45·2 (39·7, 50·7)	154/348	44·3 (39·0, 49·6)	139/350	39·7 (34·6, 45·1)	114/340	33·5 (28·5, 38·8)	··	··
PCV10-type carriage	53/332	16·0 (12·2, 20·4)	55/348	15·8 (12·1, 20·1)	37/350	10·6 (7·6, 14·3)	44/340	12·9 (9·6, 17·0)	··	··
PCV13-type carriage	89/332	26·8 (22·1, 31·9)	94/348	27·0 (22·4, 32·0)	67/350	19·1 (15·2, 23·7)	66/340	19·4 (15·3, 24·0)	··	··
Serotype 3/6A/19A carriage	43/332	13·0 (9·5, 17·0)	46/348	13·2 (9·8, 17·2)	35/350	10·0 (7·1, 13·6)	24/340	7·1 (4·6, 10·3)	··	··
Non-PCV13-type carriage	80/332	24·1 (19·6, 29·1)	85/348	24·4 (20·0, 29·3)	94/350	26·9 (22·3, 31·8)	73/340	21·5 (17·2, 26·2)	··	··
**b) Prevalence of carriage at 18 or 24 months of age**										
Any pneumococcal serotype carriage	100/335	29·9 (25·0, 35·1)	110/348	31·6 (26·8, 36·8)	101/353	28·6 (24·0, 33·6)	71/340	20·9 (16·7, 25·6)	253/742	34·1 (30·7, 37·6)
PCV10-type carriage	29/335	8·7 (5·9, 12·2)	35/348	10·1 (7·1, 13·7)	21/353	5·9 (3·7, 9·0)	21/340	6·2 (3·9, 9·3)	105/742	14·2 (11·7, 16·9)
PCV13-type carriage	53/335	15·8 (12·1, 20·2)	60/348	17·2 (13·4, 21·6)	45/353	12·7 (9·5, 16·7)	29/340	8·5 (5·8, 12·0)	175/742	23·6 (20·6, 26·8)
Serotype 3/6A/19A carriage	26/335	7·8 (5·1, 11·2)	27/348	7·8 (5·2, 11·1)	26/353	7·4 (4·9, 10·6)	8/340	2·4 (1·0, 4·6)	82/742	11·1 (8·9, 13·5)
Non-PCV13-type carriage	51/335	15·2 (11·6, 19·5)	59/348	17·0 (13·2, 21·3)	60/353	17·0 (13·2, 21·3)	44/340	12·9 (9·6, 17·0)	100/742	13·5 (11·1, 16·1)

Prev. = prevalence, expressed as a percentage. * Control group participants did not provide a swab at 6 or 12 months of age, so cannot be included in this analysis. PCV = pneumococcal conjugate vaccine. PCV10 = ten-valent PCV. PCV13 = 13-valent PCV. CI = confidence interval.

Table S7: Serotype-specific carriage prevalence over time

a) Carriage prevalence of vaccine serotypes

	0+1 PCV10 (Group V)	0+1 PCV13 (Group W)	1+1 PCV10 (Group X)	1+1 PCV13 (Group Y)	Controls (Group Z)
**PCV10 serotypes**					
**6B**					
6m	1·0 (4/387)	1·3 (5/397)	0·8 (3/390)	1·0 (4/396)	··
12m	2·4 (9/371)	2·1 (8/390)	2·1 (8/378)	1·6 (6/381)	··
18m	1·7 (6/355)	2·2 (8/364)	0·6 (2/361)	0·8 (3/360)	2·7 (21/782)
24m	1·2 (4/341)	2·5 (9/356)	2·0 (7/358)	0·3 (1/350)	3·3 (25/758)
**14**					
6m	1·0 (4/387)	0	0·3 (1/390)	0·3 (1/396)	··
12m	1·3 (5/371)	0	0·5 (2/378)	0·8 (3/381)	··
18m	0·6 (2/355)	0·5 (2/364)	0·6 (2/361)	0	1·2 (9/782)
24m	0·3 (1/341)	0	0	0·3 (1/350)	0·1 (1/758)
**19F**					
6m	2·1 (8/387)	1·3 (5/397)	0·3 (1/390)	2·3 (9/396)	··
12m	1·9 (7/371)	2·6 (10/390)	1·1 (4/378)	1·0 (4/381)	··
18m	2·3 (8/355)	1·9 (7/364)	0·6 (2/361)	1·4 (5/360)	2·4 (19/782)
24m	0·9 (3/341)	0·6 (2/356)	0·3 (1/358)	1·4 (5/350)	2·5 (19/758)
**23F**					
6m	2·1 (8/387)	2·3 (9/397)	0·8 (3/390)	1·0 (4/396)	··
12m	2·7 (10/371)	3·1 (12/390)	2·4 (9/378)	2·4 (9/381)	··
18m	2·0 (7/355)	1·6 (6/364)	1·4 (5/361)	1·7 (6/360)	2·4 (19/782)
24m	1·8 (6/341)	2·2 (8/356)	0·8 (3/358)	1·1 (4/350)	2·5 (19/758)
**Other PCV10 serotypes**					
6m	0·3 (1/387)	0	0	0·5 (2/396)	··
12m	0	0	0	0	··
18m	0·3 (1/355)	0	0·3 (1/361)	0	0·6 (5/782)
24m	0	0·6 (2/356)	0·6 (2/358)	0	0·5 (4/758)
**Additional PCV13 serotypes**					
**3**					
6m	0·3 (1/387)	0	0	0	··
12m	0·5 (2/371)	0·3 (1/390)	0	0	··
18m	0	0	0	0	0·3 (2/782)
24m	0	0	0·3 (1/358)	0	0
**6A**					
6m	2·3 (9/387)	2·5 (10/397)	0·8 (3/390)	2·0 (8/396)	··
12m	3·5 (13/371)	4·1 (16/390)	1·3 (5/378)	3·4 (13/381)	··
18m	3·4 (12/355)	2·2 (8/364)	3·9 (14/361)	1·4 (5/360)	5·5 (43/782)
24m	3·2 (11/341)	2·8 (10/356)	3·1 (11/358)	0·9 (3/350)	3·0 (23/758)
**19A**					
6m	1·6 (6/387)	1·5 (6/397)	1·3 (5/390)	1·0 (4/396)	··
12m	1·1 (4/371)	1·0 (4/390)	1·1 (4/378)	0·5 (2/381)	··
18m	0·8 (3/355)	2·2 (8/364)	0·6 (2/361)	0·3 (1/360)	1·9 (15/782)
24m	1·5 (5/341)	1·4 (5/356)	1·1 (4/358)	0·6 (2/350)	1·8 (14/758)

Data are prevalence, expressed as a percentage, (n/N). ·· indicates no sample collected at 6 or 12 months from group Z. The **18 'Other PCV10 serotypes'** comprise: **12 x 9V** (1 at 6m [V], 5 at 18m [V=1, X=1, Z=3], 6 at 24m [W=1, X=2, Z=3]), **5 x 18C** (2 at 6m [Y], 1 at 18m [Z], 2 at 24m [W=1, Z=1]), and **1 x 4** (18m [Z]). PCV = pneumococcal conjugate vaccine. PCV10 = ten-valent PCV. PCV13 = 13-valent PCV.

b) Carriage prevalence of non-vaccine serotypes

	0+1 PCV10 (Group V)	0+1 PCV13 (Group W)	1+1 PCV10 (Group X)	1+1 PCV13 (Group Y)	Controls (Group Z)
**Non-vaccine serotypes**					
**11A**					
6m	0	0	0·3 (1/390)	0	··
12m	0·3 (1/371)	0·8 (3/390)	0·5 (2/378)	0·8 (3/381)	··
18m	0·6 (2/355)	0·8 (3/364)	0·6 (2/361)	0·6 (2/360)	0·6 (5/782)
24m	1·2 (4/341)	0·3 (1/356)	0·8 (3/358)	0·9 (3/350)	1·3 (10/758)
**15A**					
6m	2·1 (8/387)	2·3 (9/397)	1·5 (6/390)	1·0 (4/396)	··
12m	2·7 (10/371)	2·1 (8/390)	3·4 (13/378)	2·1 (8/381)	··
18m	2·5 (9/355)	3·0 (11/364)	3·3 (12/361)	3·3 (12/360)	2·0 (16/782)
24m	1·8 (6/341)	2·5 (9/356)	1·4 (5/358)	2·0 (7/350)	2·0 (15/758)
**15B/C**					
6m	0·8 (3/387)	0·5 (2/397)	1·0 (4/390)	0·8 (3/396)	··
12m	1·9 (7/371)	0·8 (3/390)	1·3 (5/378)	1·6 (6/381)	··
18m	3·1 (11/355)	3·0 (11/364)	3·0 (11/361)	1·7 (6/360)	2·0 (16/782)
24m	2·3 (8/341)	4·2 (15/356)	4·5 (16/358)	2·0 (7/350)	2·0 (15/758)
**23A**					
6m	1·3 (5/387)	1·0 (4/397)	1·5 (6/390)	0	··
12m	0·8 (3/371)	0·8 (3/390)	1·6 (6/378)	0·5 (2/381)	··
18m	1·4 (5/355)	1·1 (4/364)	1·4 (5/361)	1·1 (4/360)	1·3 (10/782)
24m	1·5 (5/341)	2·2 (8/356)	0·6 (2/358)	1·1 (4/350)	1·2 (9/758)
**34**					
6m	0	0·5 (2/397)	0·8 (3/390)	1·0 (4/396)	··
12m	0·5 (2/371)	0	0·8 (3/378)	1·0 (4/381)	··
18m	0·6 (2/355)	0·8 (3/364)	0·6 (2/361)	0·8 (3/360)	0·4 (3/782)
24m	1·5 (5/341)	0	0·8 (3/358)	0·9 (3/350)	0·7 (5/758)
**Other non-vaccine serotypes**					
6m	0.5 (2/387)	1.0 (4/397)	1.5 (6/390)	2.3 (9/396)	··
12m	1.6 (6/371)	1.0 (4/390)	1.9 (7/378)	0.5 (2/381)	··
18m	0.3 (1/355)	1.6 (6/364)	0.8 (3/361)	0.0 (0/360)	0.4 (3/782)
24m	0.9 (3/341)	1.1 (4/356)	0.6 (2/358)	0.6 (2/350)	0.1 (1/758)

Data are prevalence, expressed as a percentage, (n/N). ·· indicates no sample collected at 6 or 12 months from group Z. Serotypes 15B and 15C were reported as 15B/C as they are known to interconvert, and '11F-like' was reported as 11A. The **65 'Other non-vaccine serotypes'** comprise: **17 x 35B** (3 at 6m [W=1, Y=2], 5 at 12m [V=4, X=1], 4 at 18m [V=1, W=2, Z=1], 5 at 24m [V=1, W=1, X=1, Y=2]); **12 x 13** (2 at 6m [X=1, Y=1], 3 at 12m [W=1, X=1, Y=1], 4 at 18m [W=1, X=2, Z=1], 3 at 24m [V=1, W=1, X=1]); **4 x 6C**, **16F**, and **19B** (**6C**: 2 at 6m [V], 1 at 18m [W], 1 at 24m [V], **16F**: 1 at 6m [W], 1 at 18m [W], 2 at 24m [W=1, Z=1], **19B**: 3 at 6m [Y], 1 at 12m [Y]); **3 x 7C**, **17F**, and **35A** (**7C**: 1 at 6m [W], 2 at 12m [W=1, X=1], **17F**: 1 at 6m [X], 2 at 12m [V=1, X=1], **35A**: 2 at 18m [X=1, Z=1], 1 at 24m [W]); **2 x 9A**, **19C**, **20**, and **45** (**9A**: 2 at 6m [W=1, Y=1], **19C**: 1 at 6m [Y], 1 at 12m [W], **20**: 2 at 12m [V=1, X=1], **45**: 1 at 6m [X], 1 at 12m [W]); and **1 x 8**, **10A**, **11**, **19**, **22F**, **35**, **35F** (**8**: 6m [X], **10A**: 6m [X], **11**: 12m [X], **19**: 12m [X], **22F**: 18m [W], **35**: 6m [X], **35F**: 6m [Y]). One Group W participant carried two Other non-vaccine serotypes (16F and 35B) at 24 months. PCV = pneumococcal conjugate vaccine. PCV10 = ten-valent PCV. PCV13 = 13-valent PCV.

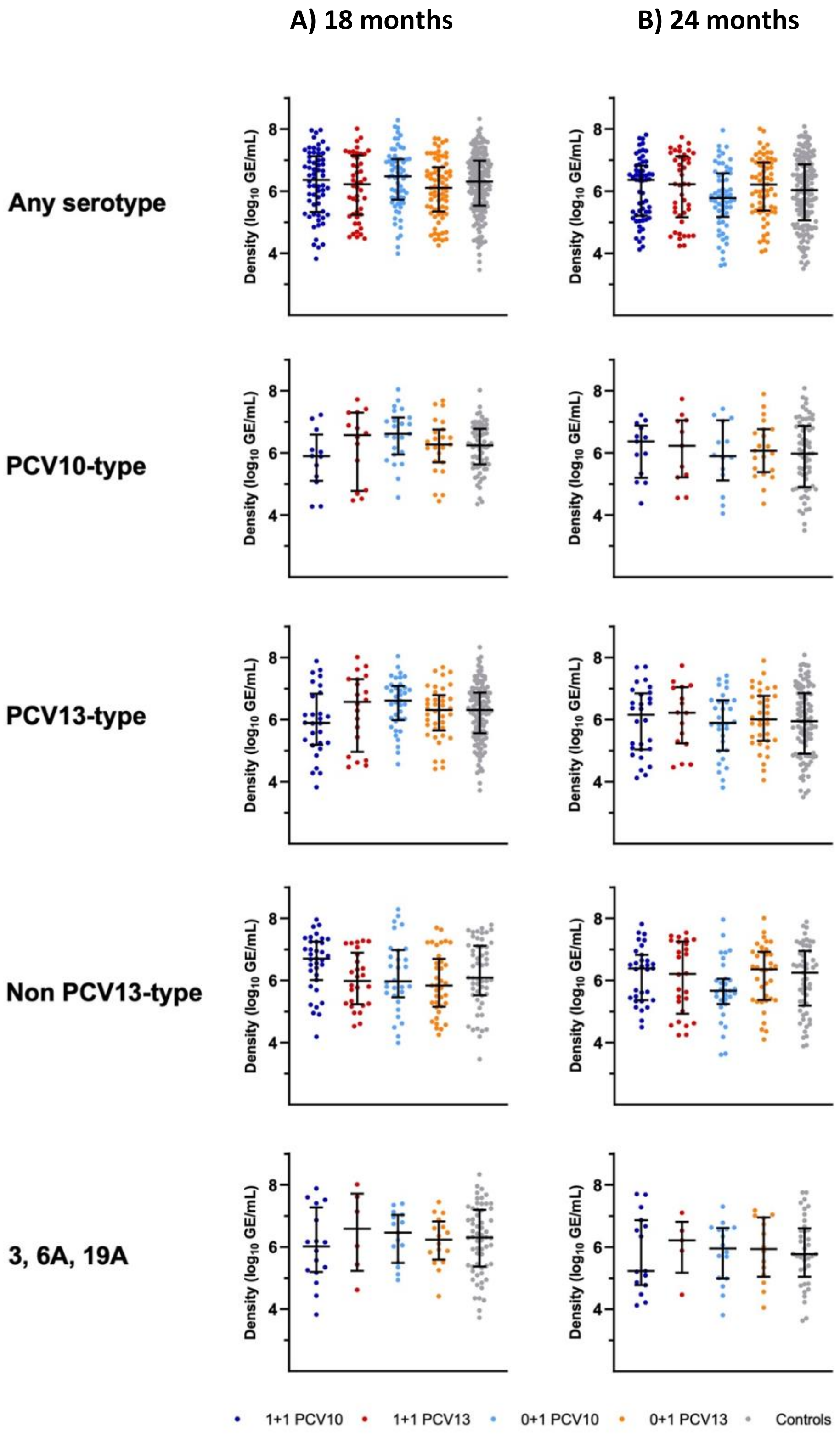

Figure S1: Pneumococcal carriage density

Scatter plots of density of carriage of any pneumococcal serotype, PCV10 serotypes, PCV13 serotypes, non-PCV13 serotypes, and serotypes 3/6A/19A ($\log_{10}$ genome equivalents per ml), among pneumococcal carriers at 18 and 24 months of age. Circles indicate data points, and solid bars indicate the median and interquartile range. PCV = pneumococcal conjugate vaccine. PCV10 = ten-valent PCV. PCV13 = 13-valent PCV.

Table S8: Number of blood samples analysed for IgG and OPA

The first 200 participants per group (Groups V-Y) and a randomly selected 200 of the first 450 Group Z participants contribute to the immunological endpoints. Immunology participants from Groups V-Y were randomised 1:3 to provide a sample either at 2/3 months or at 24 months of age in addition to samples pre- and post-12m PCV, such that each participant provides a maximum of three blood samples. Post-12m PCV samples were collected either 7 days or 28 days post-PCV, with only the 28 days post-PCV samples included in these analyses. 4-weeks post-2m PCV and 4-weeks post-12m PCV samples were excluded from analyses if they were collected less than 27 days or more than 43 days post-PCV (n=0 post-2m PCV, n=5 [n=2 for OPA] post-12m PCV). Immunology participants from Group Z provide one blood sample at 24 months of age, with control group data sourced from Groups V and W combined at the other timepoints. OPAs were conducted in a subset of samples, indicated in brackets.

	**0+1 PCV10 (Group V)**	**0+1 PCV13 (Group W)**	**1+1 PCV10 (Group X)**	**1+1 PCV13 (Group Y)**	**Controls***
2 months of age	46	-	-	-	-
3 months of age / 4-weeks post-2m PCV	-	51	50	49	97
Pre-12m PCV	186 (49)	197 (0)	189 (47-48)†	192 (41-44) †	383 (49)
4-weeks post-12m PCV	94 (46-48)	91 (48-49)	90 (46)	91 (45)	-
24 months of age	130	131	130	128	164

*Control group data sourced from the two 0+1 groups (Groups V and W combined) at 3 months (blood sample collected at 2 months in Group V) and at 12 months, and from Group Z at 24 months. Pre-12m PCV control group OPA data sourced from Group V only. †Insufficient blood volume to test all serotypes by OPA. PCV = pneumococcal conjugate vaccine. PCV10 = ten-valent PCV. PCV13 = 13-valent PCV. OPA = opsonophagocytic assay.

Table S9: Geometric Mean Concentration (95% CI) of serotype-specific IgG over time

	0+1 PCV10	0+1 PCV13	1+1 PCV10	1+1 PCV13	Controls*
**PCV10 serotypes**					
**1**					
3m	0·21 (0·17, 0·27)	0·20 (0·17, 0·24)	1·14 (0·87, 1·49)	0·54 (0·43, 0·67)	0·21 (0·18, 0·24)
12m	0·20 (0·18, 0·22)	0·20 (0·18, 0·23)	0·45 (0·40, 0·51)	0·34 (0·30, 0·38)	0·20 (0·19, 0·22)
13m	1·80 (1·51, 2·16)	3·82 (3·15, 4·63)	4·99 (4·05, 6·14)	16·45 (13·33, 20·30)	-
24m	0·60 (0·52, 0·69)	0·87 (0·76, 1·01)	0·81 (0·68, 0·95)	2·09 (1·77, 2·47)	0·31 (0·27, 0·35)
**4**					
3m	0·15 (0·12, 0·19)	0·12 (0·10, 0·15)	0·55 (0·42, 0·70)	0·39 (0·30, 0·49)	0·13 (0·11, 0·15)
12m	0·17 (0·16, 0·19)	0·18 (0·16, 0·20)	0·53 (0·48, 0·60)	0·36 (0·32, 0·42)	0·18 (0·16, 0·19)
13m	3·93 (3·32, 4·66)	3·38 (2·75, 4·16)	11·25 (9·27, 13·66)	16·71 (13·43, 20·78)	-
24m	1·00 (0·87, 1·15)	0·91 (0·79, 1·05)	1·37 (1·17, 1·61)	1·95 (1·68, 2·26)	0·28 (0·25, 0·32)
**5**					
3m	0·29 (0·23, 0·37)	0·26 (0·21, 0·31)	0·82 (0·64, 1·03)	0·47 (0·40, 0·55)	0·27 (0·24, 0·32)
12m	0·64 (0·58, 0·70)	0·66 (0·61, 0·72)	0·78 (0·71, 0·85)	0·85 (0·77, 0·93)	0·65 (0·61, 0·69)
13m	1·29 (1·15, 1·45)	1·96 (1·67, 2·28)	2·97 (2·52, 3·48)	7·30 (6·32, 8·44)	-
24m	0·99 (0·88, 1·11)	1·41 (1·25, 1·60)	1·17 (1·02, 1·34)	1·53 (1·38, 1·69)	0·78 (0·70, 0·87)
**6B**					
3m	0·26 (0·20, 0·34)	0·22 (0·18, 0·26)	0·18 (0·14, 0·22)	0·18 (0·15, 0·22)	0·24 (0·20, 0·28)
12m	0·32 (0·29, 0·35)	0·32 (0·29, 0·35)	0·73 (0·64, 0·83)	0·36 (0·32, 0·41)	0·32 (0·30, 0·34)
13m	0·52 (0·44, 0·60)	0·94 (0·77, 1·15)	5·90 (4·77, 7·31)	5·47 (4·21, 7·10)	-
24m	1·41 (1·19, 1·68)	0·98 (0·83, 1·15)	1·88 (1·54, 2·29)	1·87 (1·58, 2·20)	0·50 (0·44, 0·57)
**7F**					
3m	0·11 (0·08, 0·14)	0·06 (0·05, 0·09)	0·53 (0·39, 0·72)	0·87 (0·65, 1·15)	0·08 (0·07, 0·10)
12m	0·08 (0·07, 0·09)	0·08 (0·07, 0·09)	0·45 (0·39, 0·52)	0·44 (0·39, 0·51)	0·08 (0·07, 0·09)
13m	1·55 (1·31, 1·84)	3·64 (3·00, 4·42)	2·14 (1·81, 2·54)	4·91 (4·13, 5·84)	-
24m	0·69 (0·58, 0·81)	1·17 (1·00, 1·37)	0·52 (0·44, 0·61)	0·87 (0·75, 0·99)	0·12 (0·10, 0·15)
**9V**					
3m	0·28 (0·21, 0·37)	0·21 (0·17, 0·26)	0·41 (0·32, 0·52)	0·28 (0·23, 0·34)	0·24 (0·20, 0·28)
12m	0·30 (0·27, 0·34)	0·31 (0·27, 0·35)	0·60 (0·54, 0·67)	0·44 (0·39, 0·50)	0·30 (0·28, 0·33)
13m	1·08 (0·93, 1·26)	2·76 (2·29, 3·32)	4·24 (3·55, 5·06)	10·79 (9·19, 12·67)	-
24m	0·87 (0·75, 1·01)	1·07 (0·93, 1·23)	0·95 (0·84, 1·09)	1·60 (1·40, 1·82)	0·43 (0·38, 0·50)
**14**					
3m	0·68 (0·52, 0·91)	0·71 (0·52, 0·97)	0·75 (0·51, 1·11)	0·57 (0·45, 0·71)	0·70 (0·57, 0·86)
12m	0·45 (0·40, 0·52)	0·44 (0·38, 0·50)	1·28 (1·11, 1·48)	0·87 (0·76, 1·00)	0·44 (0·40, 0·49)
13m	1·10 (0·89, 1·36)	2·33 (1·89, 2·87)	14·76 (11·17, 19·51)	23·36 (16·98, 32·12)	-
24m	2·14 (1·81, 2·54)	3·43 (2·90, 4·06)	3·06 (2·55, 3·67)	4·82 (4·06, 5·73)	0·81 (0·68, 0·95)
**18C**					
3m	0·36 (0·30, 0·44)	0·26 (0·22, 0·31)	0·42 (0·33, 0·53)	0·50 (0·41, 0·61)	0·30 (0·27, 0·35)
12m	0·15 (0·13, 0·16)	0·14 (0·13, 0·16)	0·26 (0·23, 0·29)	0·24 (0·22, 0·27)	0·14 (0·13, 0·16)
13m	7·16 (5·95, 8·62)	4·35 (3·54, 5·35)	5·94 (5·00, 7·05)	6·07 (5·12, 7·19)	-
24m	1·26 (1·08, 1·46)	0·73 (0·64, 0·84)	1·01 (0·87, 1·17)	0·87 (0·77, 0·99)	0·25 (0·21, 0·29)
**19F**					
3m	0·47 (0·35, 0·62)	0·39 (0·31, 0·48)	0·84 (0·66, 1·07)	0·62 (0·49, 0·77)	0·42 (0·36, 0·51)
12m	0·59 (0·53, 0·64)	0·59 (0·54, 0·66)	1·62 (1·41, 1·86)	1·03 (0·90, 1·18)	0·59 (0·55, 0·63)
13m	2·70 (2·18, 3·34)	2·78 (2·16, 3·59)	54·56 (43·25, 68·81)	47·41 (38·58, 58·25)	-
24m	4·46 (3·69, 5·39)	2·21 (1·87, 2·60)	9·70 (7·88, 11·95)	5·37 (4·54, 6·36)	1·02 (0·88, 1·18)
**23F**					
3m	0·22 (0·16, 0·30)	0·20 (0·16, 0·25)	0·22 (0·17, 0·29)	0·18 (0·15, 0·22)	0·21 (0·17, 0·25)
12m	0·25 (0·23, 0·28)	0·26 (0·23, 0·29)	0·42 (0·37, 0·48)	0·29 (0·26, 0·33)	0·26 (0·24, 0·28)
13m	0·52 (0·43, 0·61)	1·26 (0·99, 1·60)	3·72 (3·03, 4·57)	6·39 (5·24, 7·79)	-
24m	1·00 (0·83, 1·21)	1·14 (0·96, 1·37)	0·99 (0·84, 1·18)	1·54 (1·27, 1·86)	0·46 (0·40, 0·54)
**Additional PCV13 serotypes**					
**3**					
3m	0·10 (0·08, 0·13)	0·10 (0·08, 0·11)	0·09 (0·07, 0·11)	0·65 (0·54, 0·78)	0·10 (0·09, 0·11)
12m	0·29 (0·26, 0·31)	0·27 (0·25, 0·31)	0·23 (0·21, 0·26)	0·40 (0·36, 0·44)	0·28 (0·26, 0·30)
13m	0·26 (0·22, 0·29)	2·62 (2·18, 3·15)	0·21 (0·18, 0·25)	4·01 (3·48, 4·61)	-
24m	0·41 (0·35, 0·48)	0·86 (0·75, 0·99)	0·37 (0·31, 0·43)	0·83 (0·73, 0·94)	0·38 (0·33, 0·45)
**6A**					
3m	0·61 (0·46, 0·81)	0·71 (0·53, 0·94)	0·60 (0·44, 0·82)	0·67 (0·51, 0·88)	0·66 (0·54, 0·80)
12m	0·59 (0·53, 0·65)	0·62 (0·55, 0·70)	0·70 (0·63, 0·79)	0·77 (0·67, 0·90)	0·60 (0·56, 0·65)
13m	0·70 (0·60, 0·82)	1·36 (1·13, 1·65)	1·46 (1·19, 1·79)	23·27 (17·95, 30·16)	-
24m	1·18 (1·00, 1·40)	1·98 (1·65, 2·38)	1·16 (0·97, 1·39)	4·78 (4·03, 5·66)	0·86 (0·76, 0·99)
**19A**					
3m	0·47 (0·35, 0·64)	0·37 (0·29, 0·48)	0·35 (0·28, 0·44)	0·37 (0·30, 0·47)	0·42 (0·35, 0·51)
12m	0·54 (0·49, 0·60)	0·59 (0·52, 0·66)	0·60 (0·53, 0·67)	0·73 (0·65, 0·82)	0·56 (0·52, 0·61)
13m	0·84 (0·71, 0·99)	3·23 (2·60, 4·01)	2·25 (1·77, 2·85)	15·97 (13·38, 19·06)	-
24m	1·44 (1·20, 1·73)	1·92 (1·59, 2·32)	1·60 (1·28, 2·00)	2·45 (2·06, 2·92)	0·91 (0·79, 1·05)

*Control group data sourced from the two 0+1 groups (Groups V and W combined) at 3 months (blood sample collected at 2 months in Group V) and at 12 months, and from Group Z at 24 months. PCV = pneumococcal conjugate vaccine. PCV10 = ten-valent PCV. PCV13 = 13-valent PCV. CI = confidence interval.

Table S10: Ratio of GMCs (95% CI) comparing vaccinated and unvaccinated participants

	1+1 PCV10 / unvaccinated			1+1 PCV13 / unvaccinated			0+1 PCV10 / unvaccinated	0+1 PCV13 / unvaccinated
	3m	12m	24m	3m	12m	24m	24m	24m
**PCV10 serotypes**								
1	5·55 (4·18, 7·36)	2·23 (1·94, 2·57)	2·64 (2·14, 3·26)	2·63 (2·03, 3·42)	1·68 (1·47, 1·92)	6·83 (5·52, 8·46)	1·96 (1·61, 2·39)	2·86 (2·35, 3·48)
4	4·11 (3·12, 5·42)	3·00 (2·61, 3·44)	4·88 (3·97, 6·00)	2·91 (2·21, 3·83)	2·04 (1·76, 2·37)	6·93 (5·68, 8·46)	3·55 (2·92, 4·32)	3·25 (2·67, 3·95)
5	2·98 (2·30, 3·85)	1·19 (1·07, 1·33)	1·50 (1·27, 1·78)	1·70 (1·35, 2·14)	1·30 (1·17, 1·45)	1·96 (1·69, 2·29)	1·27 (1·08, 1·49)	1·81 (1·54, 2·14)
6B	0·75 (0·57, 0·99)	2·28 (2·00, 2·61)	3·75 (2·97, 4·73)	0·77 (0·59, 1·00)	1·14 (1·00, 1·29)	3·73 (3·02, 4·61)	2·82 (2·27, 3·50)	1·96 (1·59, 2·41)
7F	6·43 (4·43, 9·32)	5·71 (4·71, 6·91)	4·21 (3·32, 5·33)	10·48 (7·29, 15·06)	5·60 (4·62, 6·78)	6·98 (5·57, 8·73)	5·54 (4·37, 7·02)	9·46 (7·50, 11·92)
9V	1·70 (1·27, 2·29)	1·98 (1·73, 2·27)	2·20 (1·82, 2·67)	1·17 (0·88, 1·54)	1·44 (1·24, 1·66)	3·69 (3·04, 4·48)	2·01 (1·64, 2·46)	2·47 (2·03, 3·01)
14	1·08 (0·72, 1·61)	2·89 (2·44, 3·42)	3·79 (2·97, 4·84)	0·82 (0·59, 1·14)	1·96 (1·66, 2·32)	5·98 (4·71, 7·59)	2·66 (2·09, 3·37)	4·25 (3·36, 5·39)
18C	1·39 (1·09, 1·77)	1·79 (1·57, 2·04)	4·09 (3·31, 5·05)	1·65 (1·31, 2·06)	1·68 (1·48, 1·92)	3·54 (2·90, 4·32)	5·09 (4·11, 6·30)	2·97 (2·41, 3·66)
19F	1·99 (1·48, 2·67)	2·74 (2·39, 3·14)	9·53 (7·44, 12·21)	1·45 (1·08, 1·93)	1·75 (1·53, 2·00)	5·28 (4·23, 6·59)	4·38 (3·47, 5·54)	2·17 (1·74, 2·70)
23F	1·05 (0·76, 1·46)	1·64 (1·43, 1·89)	2·15 (1·71, 2·69)	0·87 (0·64, 1·17)	1·15 (0·99, 1·32)	3·32 (2·62, 4·22)	2·17 (1·71, 2·74)	2·48 (1·97, 3·11)
**Additional PCV13 serotypes**								
3	0·87 (0·69, 1·09)	0·84 (0·74, 0·96)	0·96 (0·76, 1·21)	6·49 (5·19, 8·11)	1·42 (1·26, 1·61)	2·15 (1·73, 2·66)	1·06 (0·84, 1·33)	2·23 (1·79, 2·78)
6A	0·92 (0·64, 1·30)	1·16 (1·02, 1·33)	1·34 (1·08, 1·67)	1·01 (0·72, 1·42)	1·28 (1·10, 1·49)	5·53 (4·48, 6·82)	1·37 (1·11, 1·69)	2·29 (1·84, 2·85)
19A	0·84 (0·62, 1·15)	1·06 (0·92, 1·21)	1·75 (1·36, 2·26)	0·89 (0·66, 1·22)	1·29 (1·13, 1·48)	2·68 (2·15, 3·35)	1·58 (1·26, 1·98)	2·10 (1·67, 2·66)

Table S11: Ratio of GMCs (95% CI) comparing PCV10- and PCV13-vaccinated participants

	1+1 PCV10 / 1+1 PCV13				0+1 PCV10 / 0+1 PCV13	
	3m	12m	13m	24m	13m	24m
**PCV10 serotypes**						
1	2·11 (1·49, 2·98)	1·33 (1·12, 1·57)	0·30 (0·23, 0·41)	0·39 (0·31, 0·49)	0·47 (0·36, 0·61)	0·69 (0·56, 0·84)
4	1·41 (1·00, 2·00)	1·47 (1·23, 1·76)	0·67 (0·50, 0·90)	0·70 (0·57, 0·87)	1·16 (0·89, 1·52)	1·09 (0·90, 1·34)
5	1·75 (1·31, 2·33)	0·92 (0·80, 1·04)	0·41 (0·33, 0·50)	0·76 (0·65, 0·90)	0·66 (0·55, 0·80)	0·70 (0·59, 0·83)
6B	0·98 (0·73, 1·33)	2·01 (1·68, 2·39)	1·08 (0·77, 1·51)	1·01 (0·78, 1·30)	0·55 (0·43, 0·71)	1·44 (1·14, 1·82)
7F	0·61 (0·41, 0·93)	1·02 (0·84, 1·24)	0·44 (0·34, 0·55)	0·60 (0·49, 0·75)	0·43 (0·33, 0·55)	0·59 (0·47, 0·73)
9V	1·46 (1·07, 1·99)	1·38 (1·17, 1·62)	0·39 (0·31, 0·50)	0·60 (0·50, 0·72)	0·39 (0·31, 0·50)	0·81 (0·66, 1·00)
14	1·32 (0·84, 2·06)	1·47 (1·20, 1·80)	0·63 (0·41, 0·96)	0·63 (0·49, 0·81)	0·47 (0·35, 0·63)	0·62 (0·49, 0·79)
18C	0·84 (0·63, 1·14)	1·06 (0·91, 1·24)	0·98 (0·77, 1·24)	1·15 (0·95, 1·40)	1·65 (1·25, 2·17)	1·71 (1·39, 2·11)
19F	1·37 (0·99, 1·89)	1·56 (1·29, 1·90)	1·15 (0·85, 1·57)	1·81 (1·38, 2·36)	0·97 (0·70, 1·35)	2·02 (1·58, 2·59)
23F	1·22 (0·86, 1·71)	1·43 (1·20, 1·71)	0·58 (0·44, 0·77)	0·65 (0·50, 0·83)	0·41 (0·31, 0·55)	0·87 (0·68, 1·13)
**Additional PCV13 serotypes**						
3	0·13 (0·10, 0·17)	0·59 (0·51, 0·69)	0·05 (0·04, 0·07)	0·45 (0·36, 0·55)	0·10 (0·08, 0·12)	0·47 (0·38, 0·58)
6A	0·90 (0·60, 1·36)	0·91 (0·76, 1·09)	0·06 (0·05, 0·09)	0·24 (0·19, 0·31)	0·52 (0·40, 0·66)	0·60 (0·47, 0·76)
19A	0·94 (0·69, 1·30)	0·82 (0·69, 0·97)	0·14 (0·11, 0·19)	0·65 (0·49, 0·87)	0·26 (0·20, 0·34)	0·75 (0·58, 0·98)

Table S12: Percentage of participants (95% CI) with serotype-specific IgG ≥0.35µg/ml over time

	0+1 PCV10	0+1 PCV13	1+1 PCV10	1+1 PCV13	Controls*
**PCV10 serotypes**					
**1**					
3m	28·3 (16·0, 43·5)	15·7 (7·0, 28·6)	92·0 (80·8, 97·8)[1, 3]	71·4 (56·7, 83·4)[2]	21·6 (13·9, 31·2)
12m	23·1 (17·3, 29·8)	22·3 (16·7, 28·8)	63·0 (55·7, 69·9)[1, 3]	46·4 (39·1, 53·7)[2]	22·7 (18·6, 27·2)
13m	96·8 (91·0, 99·3)	97·8 (92·3, 99·7)	98·9 (94·0, 100)	100 (96·0, 100)	-
24m	76·9 (68·7, 83·9)	88·5 (81·8, 93·4)	80·8 (72·9, 87·2)	96·9 (92·2, 99·1)	36·0 (28·6, 43·8)
**4**					
3m	13·0 (4·9, 26·3)	9·8 (3·3, 21·4)	66·0 (51·2, 78·8)[1]	55·1 (40·2, 69·3)[2]	11·3 (5·8, 19·4)
12m	19·4 (13·9, 25·8)	19·8 (14·5, 26·1)	68·8 (61·7, 75·3)[1, 3]	49·0 (41·7, 56·3)[2]	19·6 (15·7, 23·9)
13m	100 (96·2, 100)	97·8 (92·3, 99·7)	100 (96·0, 100)	100 (96·0, 100)	-
24m	93·8 (88·2, 97·3)	90·8 (84·5, 95·2)	94·6 (89·2, 97·8)	99·2 (95·7, 100)	34·8 (27·5, 42·6)
**5**					
3m	30·4 (17·7, 45·8)	29·4 (17·5, 43·8)	90·0 (78·2, 96·7)[1, 3]	65·3 (50·4, 78·3)[2]	29·9 (21·0, 40·0)
12m	84·4 (78·4, 89·3)	86·8 (81·3, 91·2)	88·9 (83·5, 93·0)	91·1 (86·2, 94·8)	85·6 (81·7, 89·0)
13m	100 (96·2, 100)	100 (96·0, 100)	100 (96·0, 100)	100 (96·0, 100)	-
24m	92·3 (86·3, 96·2)	98·5 (94·6, 99·8)	95·4 (90·2, 98·3)	98·4 (94·5, 99·8)	87·2 (81·1, 91·9)
**6B**					
3m	30·4 (17·7, 45·8)	25·5 (14·3, 39·6)	20·0 (10·0, 33·7)	22·4 (11·8, 36·6)	27·8 (19·2, 37·9)
12m	48·9 (41·5, 56·3)	43·7 (36·6, 50·9)	85·2 (79·3, 89·9)[1, 3]	48·4 (41·2, 55·7)	46·2 (41·1, 51·4)
13m	64·9 (54·4, 74·5)	90·1 (82·1, 95·4)	98·9 (94·0, 100)	98·9 (94·0, 100)	-
24m	94·6 (89·2, 97·8)	86·3 (79·2, 91·6)	97·7 (93·4, 99·5)	96·1 (91·1, 98·7)	59·1 (51·2, 66·7)
**7F**					
3m	8·7 (2·4, 20·8)	2·0 (0·0, 10·4)	62·0 (47·2, 75·3)[1]	85·7 (72·8, 94·1)[2, 3]	5·2 (1·7, 11·6)
12m	9·7 (5·8, 14·9)	8·1 (4·7, 12·9)	58·7 (51·4, 65·8)[1]	58·9 (51·5, 65·9)[2]	8·9 (6·2, 12·2)
13m	95·7 (89·5, 98·8)	97·8 (92·3, 99·7)	100 (96·0, 100)	98·9 (94·0, 100)	-
24m	76·2 (67·9, 83·2)	93·1 (87·4, 96·8)	65·4 (56·5, 73·5)	89·8 (83·3, 94·5)	18·3 (12·7, 25·1)
**9V**					
3m	32·6 (19·5, 48·0)	25·5 (14·3, 39·6)	52·0 (37·4, 66·3)[1, 3]	26·5 (14·9, 41·1)	28·9 (20·1, 39·0)
12m	44·1 (36·8, 51·5)	43·1 (36·1, 50·4)	76·2 (69·5, 82·1)[1, 3]	58·9 (51·5, 65·9)[2]	43·6 (38·6, 48·7)
13m	94·7 (88·0, 98·3)	98·9 (94·0, 100)	100 (96·0, 100)	100 (96·0, 100)	-
24m	86·9 (79·9, 92·2)	91·6 (85·5, 95·7)	91·5 (85·4, 95·7)	96·9 (92·2, 99·1)	57·9 (50·0, 65·6)
**14**					
3m	78·3 (63·6, 89·1)	78·4 (64·7, 88·7)	78·0 (64·0, 88·5)	79·6 (65·7, 89·8)	78·4 (68·8, 86·1)
12m	63·4 (56·1, 70·4)	59·4 (52·2, 66·3)	91·5 (86·6, 95·1)[1]	86·5 (80·8, 91·0)[2]	61·4 (56·3, 66·3)
13m	92·6 (85·3, 97·0)	100 (96·0, 100)	97·8 (92·2, 99·7)	100 (96·0, 100)	-
24m	98·5 (94·6, 99·8)	100 (97·2, 100)	99·2 (95·8, 100)	100 (97·2, 100)	79·3 (72·3, 85·2)
**18C**					
3m	50·0 (34·9, 65·1)	27·5 (15·9, 41·7)	56·0 (41·3, 70·0)	75·5 (61·1, 86·7)[2]	38·1 (28·5, 48·6)
12m	12·9 (8·4, 18·6)	12·7 (8·4, 18·2)	32·3 (25·7, 39·4)[1]	30·7 (24·3, 37·8)[2]	12·8 (9·6, 16·6)
13m	100 (96·2, 100)	100 (96·0, 100)	100 (96·0, 100)	100 (96·0, 100)	-
24m	94·6 (89·2, 97·8)	82·4 (74·8, 88·5)	92·3 (86·3, 96·2)	91·4 (85·1, 95·6)	31·7 (24·7, 39·4)
**19F**					
3m	56·5 (41·1, 71·1)	52·9 (38·5, 67·1)	86·0 (73·3, 94·2)[1]	79·6 (65·7, 89·8)[2]	54·6 (44·2, 64·8)
12m	75·8 (69·0, 81·8)	75·6 (69·0, 81·5)	97·9 (94·7, 99·4)[1]	94·8 (90·6, 97·5)[2]	75·7 (71·1, 79·9)
13m	100 (96·2, 100)	97·8 (92·3, 99·7)	100 (96·0, 100)	100 (96·0, 100)	-
24m	100 (97·2, 100)	99·2 (95·8, 100)	100 (97·2, 100)	100 (97·2, 100)	89·6 (83·9, 93·8)
**23F**					
3m	32·6 (19·5, 48·0)	19·6 (9·8, 33·1)	32·0 (19·5, 46·7)	16·3 (7·3, 29·7)	25·8 (17·4, 35·7)
12m	34·9 (28·1, 42·3)	34·5 (27·9, 41·6)	60·8 (53·5, 67·8)[1, 3]	36·5 (29·6, 43·7)	34·7 (30·0, 39·7)
13m	69·1 (58·8, 78·3)	87·9 (79·4, 93·8)	100 (96·0, 100)	100 (96·0, 100)	-
24m	84·6 (77·2, 90·3)	85·5 (78·3, 91·0)	89·2 (82·6, 94·0)	90·6 (84·2, 95·1)	57·3 (49·4, 65·0)
**Additional PCV13 serotypes**					
**3**					
3m	8·7 (2·4, 20·8)	2·0 (0·0, 10·4)	4·0 (0·5, 13·7)	79·6 (65·7, 89·8)[2, 3]	5·2 (1·7, 11·6)
12m	39·2 (32·2, 46·7)	36·5 (29·8, 43·7)	31·7 (25·2, 38·9)	55·7 (48·4, 62·9)[2, 3]	37·9 (33·0, 42·9)
13m	37·2 (27·5, 47·8)	96·7 (90·7, 99·3)	26·7 (17·9, 37·0)	100 (96·0, 100)	-
24m	50·0 (41·1, 58·9)	90·1 (83·6, 94·6)	46·2 (37·4, 55·1)	90·6 (84·2, 95·1)	51·2 (43·3, 59·1)
**6A**					
3m	78·3 (63·6, 89·1)	74·5 (60·4, 85·7)	70·0 (55·4, 82·1)	71·4 (56·7, 83·4)	76·3 (66·6, 84·3)
12m	79·0 (72·5, 84·6)	72·6 (65·8, 78·7)	82·0 (75·8, 87·2)	81·3 (75·0, 86·5)	75·7 (71·1, 79·9)
13m	81·9 (72·6, 89·1)	93·4 (86·2, 97·5)	92·2 (84·6, 96·8)	100 (96·0, 100)	-
24m	90·0 (83·5, 94·6)	98·5 (94·6, 99·8)	91·5 (85·4, 95·7)	100 (97·2, 100)	89·6 (83·9, 93·8)
**19A**					
3m	56·5 (41·1, 71·1)	51·0 (36·6, 65·2)	56·0 (41·3, 70·0)	51·0 (36·3, 65·6)	53·6 (43·2, 63·8)
12m	75·3 (68·4, 81·3)	72·1 (65·3, 78·2)	73·0 (66·1, 79·2)	80·2 (73·9, 85·6)	73·6 (68·9, 78·0)
13m	92·6 (85·3, 97·0)	98·9 (94·0, 100)	96·7 (90·6, 99·3)	100 (96·0, 100)	-
24m	96·9 (92·3, 99·2)	98·5 (94·6, 99·8)	96·2 (91·3, 98·7)	96·9 (92·2, 99·1)	86·0 (79·7, 90·9)

*Control group data sourced from the two 0+1 groups (Groups V and W combined) at 3 months (blood sample collected at 2 months in Group V) and at 12 months, and from Group Z at 24 months. PCV = pneumococcal conjugate vaccine. PCV10 = ten-valent PCV. PCV13 = 13-valent PCV. CI = confidence interval.

Table S13: Difference (95% CI) in percentage of participants achieving serotype-specific IgG ≥0.35µg/ml and OI ≥8 after a 2-month dose of PCV, comparing vaccinated and unvaccinated participants, and comparing PCV10- and PCV13-recipients

	1+1 PCV10 - unvaccinated			1+1 PCV13 - unvaccinated			1+1 PCV10 - 1+1 PCV13		
	**3m IgG**	**12m IgG**	**12m OPA**	**3m IgG**	**12m IgG**	**12m OPA**	**3m IgG**	**12m IgG**	**12m OPA**
**PCV10 serotypes**									
1	70.4 (56.1, 78.9)	40.2 (31.9, 47.9)	27.2 (12.3, 41.6)	49.8 (33.2, 62.6)	23.6 (15.4, 31.7)	5.0 (-6.1, 17.4)	20.6 (5.3, 35.2)	16.6 (6.6, 26.1)	22.2 (5.6, 37.3)
4	54.7 (38.8, 67.2)	49.2 (41.1, 56.4)	31.3 (16.8, 45.5)	43.8 (27.9, 57.7)	29.4 (21.2, 37.3)	18.9 (6.0, 33.3)	10.9 (-8.1, 28.9)	19.8 (10.0, 29.1)	12.4 (-6.0, 29.4)
5	60.1 (45.1, 70.1)	3.2 (-2.9, 8.6)	60.5 (43.9, 72.9)	35.4 (18.4, 49.8)	5.5 (-0.3, 10.6)	45.7 (29.3, 60.1)	24.7 (8.3, 39.8)	-2.3 (-8.5, 3.9)	14.8 (-5.4, 33.4)
6B	-7.8 (-20.9, 7.4)	39.0 (31.3, 45.6)	43.9 (26.3, 58.2)	-5.4 (-18.9, 10.2)	2.2 (-6.4, 10.8)	-3.6 (-15.1, 8.1)	-2.4 (-18.5, 13.6)	36.7 (27.7, 45.0)	47.5 (30.2, 61.4)
7F	56.8 (41.6, 69.3)	49.9 (42.0, 57.1)	73.2 (56.1, 83.1)	80.6 (66.6, 88.3)	50.0 (42.2, 57.1)	72.4 (54.7, 82.5)	-23.7 (-39.3, -6.4)	-0.1 (-9.9, 9.7)	0.8 (-11.0, 13.3)
9V	23.1 (6.5, 38.6)	32.6 (24.3, 39.9)	62.3 (43.8, 74.5)	-2.3 (-16.5, 13.6)	15.3 (6.6, 23.6)	27.2 (8.7, 44.0)	25.5 (6.2, 42.2)	17.3 (7.9, 26.3)	35.1 (14.6, 52.0)
14	-0.4 (-15.3, 12.7)	30.2 (23.4, 36.1)	36.1 (16.3, 52.2)	1.2 (-13.7, 14.1)	25.1 (17.8, 31.6)	40.3 (20.2, 56.0)	-1.6 (-17.6, 14.5)	5.1 (-1.3, 11.5)	-4.2 (-21.6, 13.9)
18C	17.9 (0.9, 33.6)	19.5 (12.2, 27.1)	45.8 (30.7, 59.7)	37.4 (20.5, 50.8)	17.9 (10.8, 25.4)	34.1 (19.9, 48.9)	-19.5 (-36.4, -0.8)	1.5 (-7.7, 10.8)	11.7 (-8.1, 30.2)
19F	31.4 (15.9, 43.5)	22.2 (17.0, 26.9)	66.5 (48.3, 77.8)	25.0 (8.6, 38.3)	19.1 (13.3, 24.2)	40.1 (21.2, 55.8)	6.4 (-8.7, 21.4)	3.1 (-0.9, 7.4)	26.3 (6.6, 43.7)
23F	6.2 (-8.5, 22.0)	26.1 (17.5, 34.2)	34.5 (16.9, 49.7)	-9.4 (-21.8, 5.4)	1.7 (-6.4, 10.1)	9.3 (-5.5, 24.9)	15.7 (-1.3, 31.5)	24.4 (14.4, 33.7)	25.2 (5.5, 42.0)
**Additional PCV13 serotypes**									
3	-1.2 (-8.1, 8.7)	-6.1 (-14.1, 2.3)	7.1 (-12.2, 25.6)	74.4 (59.8, 83.8)	17.9 (9.2, 26.2)	43.0 (23.4, 58.1)	-75.6 (-85.0, -59.3)	-24.0 (-33.2, -14.1)	-36.0 (-51.8, -16.4)
6A	-6.3 (-21.9, 8.1)	6.3 (-1.0, 12.9)	14.7 (0.9, 28.7)	-4.9 (-20.5, 9.4)	5.5 (-1.8, 12.2)	14.3 (0.4, 28.9)	-1.4 (-18.9, 16.2)	0.8 (-7.1, 8.5)	0.4 (-16.4, 16.7)
19A	2.4 (-14.3, 18.6)	-0.6 (-8.5, 6.8)	12.9 (-4.7, 29.6)	-2.6 (-19.2, 14.1)	6.6 (-0.9, 13.4)	18.2 (-0.3, 35.5)	5.0 (-14.2, 23.6)	-7.2 (-15.6, 1.3)	-5.3 (-24.1, 13.8)

"Unvaccinated" IgG data sourced from Groups V and W combined (the two 0+1 groups). A subset of samples (n=50 per group from Groups V [unvaccinated], X [PCV10], and Y [PCV13]) were analysed by OPA. PCV = pneumococcal conjugate vaccine. PCV10 = 10-valent PCV. PCV13 = 13-valent PCV. OPA = opsonophagocytic assay. OI = opsonisation index.

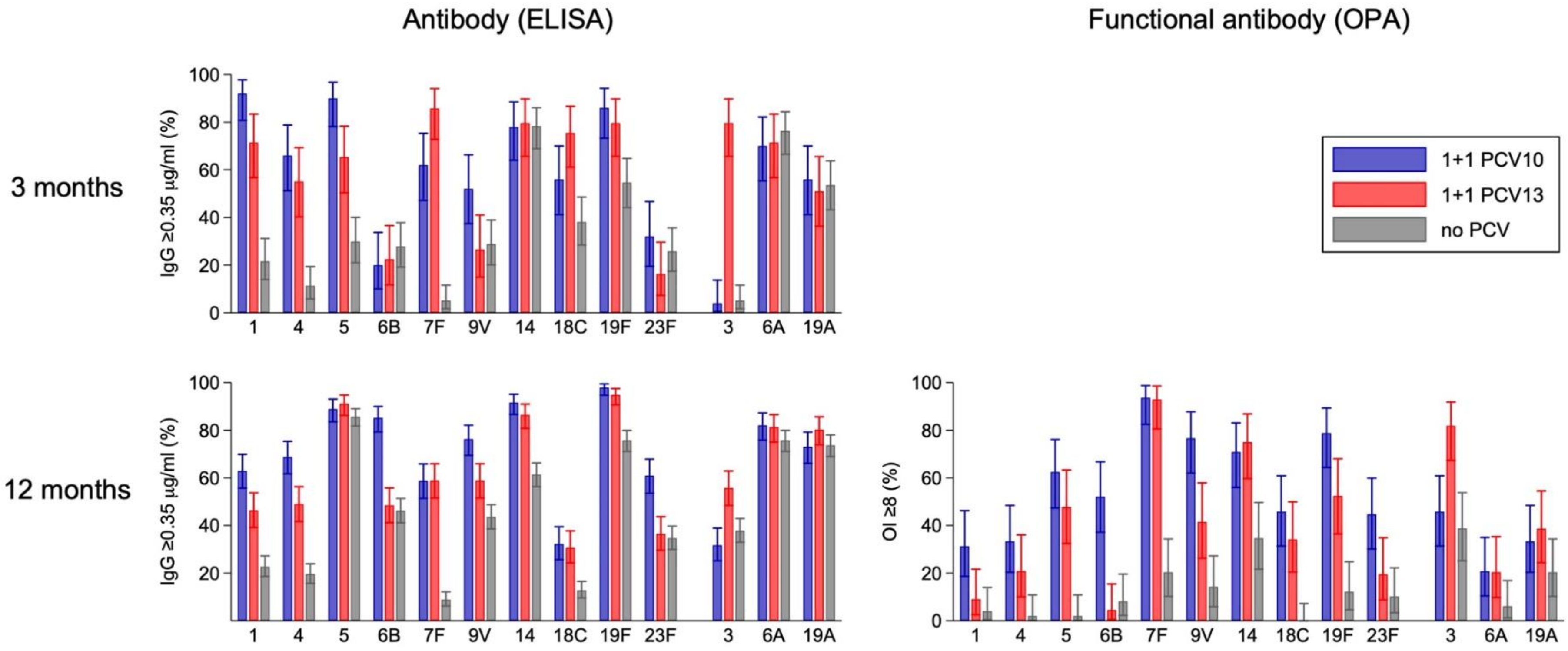

Figure S2: Protection between doses afforded by a 1+1 schedule of PCV10 or PCV13
Percentage of participants achieving protective levels of serotype-specific IgG (IgG ≥0.35μg/ml, left column) and functional antibody by OPA (OI ≥8, right column), four weeks after a 2-month dose (3 months of age, top row) and before a 12-month dose (12 months of age, bottom row) of PCV10, PCV13, or no PCV. "No PCV" ELISA data sourced from Groups V and W combined (the two 0+1 groups). A subset of samples (n=50 per group from Groups V [no PCV], X [PCV10], and Y [PCV13]) were analysed by OPA. PCV = pneumococcal conjugate vaccine. PCV10 = 10-valent PCV. PCV13 = 13-valent PCV. OPA = opsonophagocytic assay. OI = opsonisation index.

Table S14: Percentage (95% CI) of participants with opsonisation index (OI) ≥8 pre- and 4-weeks post-12m dose of PCV

	Pre-12m PCV			Post-12m PCV			
	**0+1 PCV10***	**1+1 PCV10**	**1+1 PCV13**	**0+1 PCV10**	**0+1 PCV13**	**1+1 PCV10**	**1+1 PCV13**
**PCV10 serotypes**							
1	4·1 (0·5, 14·0)	31·3 (18·7, 46·3)	9·1 (2·5, 21·7)	78·7 (64·3, 89·3)	87·5 (74·8, 95·3)	95·7 (85·2, 99·5)	100 (92·0, 100)
4	2·0 (0·1, 10·9)	33·3 (20·4, 48·4)	20·9 (10·0, 36·0)	89·6 (77·3, 96·5)	97·9 (88·9, 99·9)	100 (92·3, 100)	100 (92·0, 100)
5	2·0 (0·1, 10·9)	62·5 (47·4, 76·0)	47·7 (32·5, 63·3)	76·6 (62·0, 87·7)	91·5 (79·6, 97·6)	100 (92·3, 100)	100 (92·0, 100)
6B	8·2 (2·3, 19·6)	52·1 (37·2, 66·7)	4·5 (0·6, 15·5)	57·4 (42·2, 71·7)	85·4 (72·2, 93·9)	100 (92·3, 100)	97·7 (88·0, 99·9)
7F	20·4 (10·2, 34·3)	93·6 (82·5, 98·7)	92·9 (80·5, 98·5)	97·9 (88·7, 99·9)	97·9 (88·9, 99·9)	100 (92·3, 100)	100 (92·0, 100)
9V	14·3 (5·9, 27·2)	76·6 (62·0, 87·7)	41·5 (26·3, 57·9)	89·1 (76·4, 96·4)	97·9 (88·9, 99·9)	100 (92·3, 100)	100 (92·0, 100)
14	34·7 (21·7, 49·6)	70·8 (55·9, 83·0)	75·0 (59·7, 86·8)	95·8 (85·7, 99·5)	97·9 (88·9, 99·9)	97·8 (88·5, 99·9)	100 (92·0, 100)
18C	0·0 (0·0, 7·3)	45·8 (31·4, 60·8)	34·1 (20·5, 49·9)	97·9 (88·7, 99·9)	97·9 (88·9, 99·9)	100 (92·3, 100)	100 (92·0, 100)
19F	12·2 (4·6, 24·8)	78·7 (64·3, 89·3)	52·4 (36·4, 68·0)	93·6 (82·5, 98·7)	97·9 (88·9, 99·9)	100 (92·3, 100)	100 (92·0, 100)
23F	10·2 (3·4, 22·2)	44·7 (30·2, 59·9)	19·5 (8·8, 34·9)	65·2 (49·8, 78·6)	87·5 (74·8, 95·3)	97·8 (88·5, 99·9)	100 (92·0, 100)
**Additional PCV13 serotypes**							
3	38·8 (25·2, 53·8)	45·8 (31·4, 60·8)	81·8 (67·3, 91·8)	38·3 (24·5, 53·6)	95·8 (85·7, 99·5)	32·6 (19·5, 48·0)	100 (92·0, 100)
6A	6·1 (1·3, 16·9)	20·8 (10·5, 35·0)	20·5 (9·8, 35·3)	50·0 (35·2, 64·8)	91·7 (80·0, 97·7)	71·7 (56·5, 84·0)	100 (92·0, 100)
19A	20·4 (10·2, 34·3)	33·3 (20·4, 48·4)	38·6 (24·4, 54·5)	93·8 (82·8, 98·7)	100 (92·6, 100)	93·5 (82·1, 98·6)	100 (92·0, 100)

* Pre-12m PCV OPAs were only conducted in one of the two 0+1 groups, as these groups have the same vaccination status up to 12 months of age and represent unvaccinated controls at this timepoint. PCV = pneumococcal conjugate vaccine. PCV10 = ten-valent PCV. PCV13 = 13-valent PCV. OPA = opsonophagocytic assay.

Table S15: Ratio (95% CI) of geometric mean concentrations of IgG and functional antibody comparing participants who received a 1+1 or 0+1 schedule of a) PCV10 and b) PCV13

a) PCV10-recipients

	Ratio of GMCs [1+1 / 0+1] (95% CI)			Ratio of GMOIs [1+1/ 0+1] (95% CI)	
	**Pre-12m PCV**	**Post-12m PCV**	**24 months of age**	**Pre-12m PCV**	**Post-12m PCV**
**PCV10 serotypes**					
1	2·23 (1·89, 2·64)	2·76 (2·10, 3·63)	1.35 (1.08, 1.67)	1·74 (1·26, 2·42)	13·32 (7·29, 24·36)
4	3·08 (2·63, 3·61)	2·86 (2·22, 3·70)	1.37 (1.11, 1.70)	1·94 (1·39, 2·69)	4·85 (2·38, 9·88)
5	1·21 (1·06, 1·38)	2·29 (1·89, 2·79)	1.18 (0.99, 1.41)	3·76 (2·70, 5·23)	36·42 (23·78, 55·79)
6B	2·27 (1·93, 2·65)	11·46 (8·84, 14·85)	1.33 (1.02, 1.73)	2·98 (1·51, 5·91)	35·97 (15·50, 83·49)
7F	5·62 (4·54, 6·96)	1·38 (1·09, 1·75)	0.76 (0.60, 0.96)	24·95 (11·78, 52·86)	1·46 (0·84, 2·54)
9V	1·99 (1·71, 2·32)	3·93 (3·12, 4·96)	1.10 (0.90, 1.34)	8·58 (4·63, 15·91)	3·69 (1·85, 7·36)
14	2·82 (2·32, 3·44)	13·42 (9·50, 18·94)	1.43 (1.11, 1.83)	5·66 (1·91, 16·76)	1·38 (0·73, 2·63)
18C	1·79 (1·54, 2·07)	0·83 (0·64, 1·07)	0.80 (0.65, 0.99)	2·28 (1·65, 3·15)	1·59 (0·90, 2·81)
19F	2·76 (2·34, 3·26)	20·23 (14·79, 27·66)	2.17 (1.64, 2.88)	12·51 (6·78, 23·08)	5·93 (3·15, 11·17)
23F	1·66 (1·41, 1·96)	7·21 (5·53, 9·40)	0.99 (0.77, 1.28)	2·18 (1·17, 4·07)	7·91 (3·03, 20·64)
**Additional PCV13 serotypes**					
3	0·82 (0·71, 0·95)	0·84 (0·67, 1·05)	0.90 (0.72, 1.14)	0·98 (0·72, 1·33)	1·01 (0·65, 1·56)
6A	1·19 (1·03, 1·39)	2·07 (1·61, 2·68)	0.98 (0.77, 1.26)	1·47 (0·76, 2·85)	3·26 (1·01, 10·53)
19A	1·10 (0·94, 1·29)	2·68 (2·01, 3·57)	1.11 (0.83, 1.48)	1·58 (1·01, 2·48)	2·32 (1·19, 4·55)

b) PCV13-recipients

	Ratio of GMCs [1+1 / 0+1] (95% CI)			Ratio of GMOIs [1+1/ 0+1] (95% CI)	
	**Pre-12m PCV**	**Post-12m PCV**	**24 months of age**	**Pre-12m PCV**	**Post-12m PCV**
**PCV10 serotypes**					
1	1·68 (1·44, 1·96)	4·30 (3·24, 5·71)	2.39 (1.92, 2.97)	1·13 (0·92, 1·39)	11·40 (6·02, 21·58)
4	1·99 (1·66, 2·38)	4·94 (3·67, 6·66)	2.14 (1.74, 2.62)	1·88 (1·21, 2·92)	3·57 (2·15, 5·94)
5	1·28 (1·13, 1·46)	3·74 (3·03, 4·61)	1.08 (0.92, 1.27)	2·78 (2·05, 3·75)	23·08 (12·74, 41·81)
6B	1·15 (0·98, 1·34)	5·83 (4·20, 8·09)	1.91 (1.52, 2.40)	0·75 (0·47, 1·20)	9·40 (3·45, 25·59)
7F	5·68 (4·57, 7·05)	1·35 (1·04, 1·75)	0.74 (0.60, 0.91)	36·01 (16·77, 77·33)	1·55 (0·95, 2·51)
9V	1·43 (1·20, 1·69)	3·91 (3·06, 5·00)	1.49 (1.23, 1.81)	2·98 (1·54, 5·77)	2·57 (1·58, 4·16)
14	2·00 (1·65, 2·43)	10·01 (6·86, 14·61)	1.41 (1.11, 1.79)	6·10 (2·07, 17·96)	2·26 (1·35, 3·79)
18C	1·69 (1·44, 1·97)	1·39 (1·07, 1·82)	1.19 (0.99, 1.44)	1·90 (1·40, 2·58)	1·81 (1·08, 3·03)
19F	1·74 (1·47, 2·06)	17·04 (12·30, 23·59)	2.43 (1.93, 3.07)	2·50 (1·67, 3·74)	7·74 (4·01, 14·92)
23F	1·14 (0·96, 1·35)	5·06 (3·72, 6·90)	1.34 (1.04, 1.74)	1·55 (0·79, 3·02)	4·71 (2·10, 10·53)
**Additional PCV13 serotypes**					
3	1·45 (1·25, 1·68)	1·53 (1·21, 1·92)	0.96 (0.80, 1.16)	2·96 (1·90, 4·59)	2·28 (1·47, 3·56)
6A	1·25 (1·04, 1·51)	17·06 (12·37, 23·51)	2.41 (1.88, 3.09)	1·38 (0·72, 2·67)	5·27 (2·56, 10·88)
19A	1·24 (1·05, 1·46)	4·95 (3·75, 6·53)	1.28 (0.98, 1.65)	1·70 (1·15, 2·51)	4·77 (2·86, 7·98)

Table S16: Serious Adverse Events

	Group V	Group W	Group X	Group Y	Group Z	Total
Total admissions, n	71	69	71	66	138	415
Reason for admission, n (%)						
Respiratory illness*	38 (54%)	27 (39%)	27 (38%)	28 (42%)	65 (47%)	185 (44%)
Diarrhoea/vomiting	5 (7%)	7 (10%)	9 (13%)	8 (11%)	25 (18%)	54 (13%)
Fever/convulsion	7 (10%)	8 (12%)	11 (15%)	5 (8%)	13 (9%)	44 (11%)
Ear-nose-throat conditions	3 (4%)	14 (20%)	5 (7%)	7 (11%)	9 (7%)	38 (9%)
Hand-foot-and-mouth disease	7 (10%)	5 (7%)	7 (10%)	7 (11%)	11 (8%)	37 (9%)
Other	11 (15%)	8 (12%)	12 (17%)	11 (17%)	15 (11%)	57 (14%)

* Respiratory illness included bronchitis, bronchiolitis, pneumonia, and asthma. The 57 other reasons for admission were: skin conditions (n=9), intussusception (n=9), urinary tract infection (n=9), dengue (n=7), accident (n=4), other (n=19). The majority of hospitalisations (99%) were unrelated to vaccination, with 1 probably related (a case of immune thrombocytopenic purpura one day after receiving 2m PCV10 and *Infanrix-hexa*) and 3 possibly related (digestive disorder one day after 3m *Infanrix-hexa*, sore throat one day after 18m *Infanrix-hexa*, cellulitis in shoulder one day after 18m *Infanrix-hexa*).

Table S17: Post-PCV reactogenicity

Symptom, n (%)	2m PCV		12m PCV			
	Group X (PCV10)	Group Y (PCV13)	Group V (PCV10)	Group W (PCV13)	Group X (PCV10)	Group Y (PCV13)
Number vaccinated	401*	400*	371	390	379	381
Number withdrawn before post-PCV visit	5	4	9	14	11	6
Number with diary card	396	396	362	375†	367†	375
Any reported symptom	93 (23·5%)	78 (19·7%)	40 (11·0%)	45 (12·0%)	31 (8·4%)	32 (8·5%)
Axillary fever >38.0°C	63 (15·9%)	52 (13·1%)	12 (3·3%)	21 (5·6%)	18 (4·9%)	19 (5·1%)
Erythema >30mm	4 (1·0%)	4 (1·0%)	7 (1·9%)	2 (0·5%)	5 (1·4%)	8 (2·1%)
Other reported symptoms						
Runny nose	1 (0·3%)	3 (0·8%)	10 (2·8%)	5 (1·3%)	6 (1·6%)	3 (0·8%)
Cough	2 (0·5%)	2 (0·5%)	8 (2·2%)	9 (2·4%)	5 (1·4%)	2 (0·5%)
Diarrhoea	3 (0·8%)	2 (0·5%)	1 (0·3%)	2 (0·5%)	3 (0·8%)	1 (0·3%)
Swelling >30mm	2 (0·5%)	0 (0%)	5 (1·4%)	1 (0·3%)	1 (0·3%)	1 (0·3%)
Other	23 (5·8%)	22 (5·6%)	5 (1·4%)	15 (4·0%)	3 (0·8%)	3 (0·8%)

Frequency (%) of reported symptoms in parent-held diary cards. Participants can report multiple symptoms. * 1 participant from each of Groups X and Y was withdrawn prior to their 2m PCV. † 1 participant from each of Groups W and X did not return their diary card. PCV = pneumococcal conjugate vaccine. PCV10 = ten-valent PCV. PCV13 = 13-valent PCV.
